# Estimating the distributional impact of improving access to snake antivenom in urban and rural Lao People’s Democratic Republic: An extended cost-effectiveness analysis

**DOI:** 10.1371/journal.pntd.0014420

**Published:** 2026-06-04

**Authors:** Chanthawat Patikorn, Thanapol Khuharatanachai, Jeong-Yeon Cho, Suthira Taychakhoonavudh, Mayfong Mayxay, Khamlub Senbounsou, Joerg Blessmann, Nathorn Chaiyakunapruk

**Affiliations:** 1 Department of Social and Administrative Pharmacy, Faculty of Pharmaceutical Sciences, Chulalongkorn University, Bangkok, Thailand; 2 Department of Pharmacotherapy, College of Pharmacy, University of Utah, Salt Lake City, Utah, United States of America; 3 Institute of Research and Education Development (IRED), University of Health Sciences, Ministry of Health, Vientiane, Lao People’s Democratic Republic; 4 Lao-Oxford-Mahosot Hospital-Wellcome Trust Research Unit, Microbiology Laboratory, Mahosot Hospital, Vientiane, Lao People’s Democratic Republic; 5 Centre for Tropical Medicine and Global Health, Nuffield Department of Medicine, University of Oxford, Oxford, United Kingdom; 6 Saw Swee Hock School of Public Health, National University of Singapore, Singapore, Singapore; 7 Research Group Neglected Diseases and Envenoming, Department of Implementation Research, Bernhard Nocht Institute for Tropical Medicine, Hamburg, Germany; 8 IDEAS Center, Veterans Affairs Salt Lake City Healthcare System, Salt Lake City, Utah, United States of America; French Guiana University Hospital, FRENCH GUIANA

## Abstract

**Introduction:**

Snakebite is a significant public health issue in the Lao People’s Democratic Republic (PDR), with victims often seeking traditional healers due to inadequate antivenom supply and high out-of-pocket (OOP) expenses, contributing to inequities between urban and rural populations.

**Methods:**

An extended cost-effectiveness analysis (ECEA) was conducted to evaluate the distributional impact of improving access to antivenom in urban and rural Lao PDR, where all victims with systemic envenoming clinically indicated for antivenom receive antivenom at conventional hospitals, on disease burdens (deaths and disability-adjusted life years [DALYs]) and economic burden (%household OOP expenditure per monthly income). Sensitivity analyses were performed.

**Results:**

Rural areas had higher mortality (3.14 vs. 0.35, +2.79 per 100,000 population), DALYs lost (78.97 vs. 8.86, +70.11 per 100,000 population), and household OOP expenses (39% vs. 38%, +1% of household monthly income) than urban areas. Full access to antivenom reduced health inequities in mortality (from +2.79 to +1.22 per 100,000 population) and DALYs (from +70.11 to +32.14 per 100,000 population) but increased inequities in household OOP expenses (from +1% to +39% of household monthly income). These differences had considerable uncertainties. Sensitivity analyses showed that free snakebite treatment and transportation costs with one caregiver reduced household OOP expenses in both areas (5% rural, 6% urban).

**Conclusions:**

While improving access to snake antivenom mitigates health inequities in disease burden, it exacerbates financial inequities between urban and rural areas. Policies targeting equitable access to care and financial protection are critical to achieving health equity for snakebites in the Lao PDR.

## Author summary

Snakebite is a significant public health issue in the Lao People’s Democratic Republic (PDR), with victims often seeking traditional healers due to inadequate antivenom supply and high out-of-pocket (OOP) healthcare expenses. These challenges contribute to health inequities between urban and rural populations. A previous cost-effectiveness analysis highlighted the substantial clinical and economic benefits of hypothetically treating all snakebite envenoming with clinical indications of receiving antivenoms in Lao PDR with antivenoms. However, the study did not examine the distributional impacts of antivenom access across urban and rural populations, nor did it evaluate the potential benefits of financial risk protection. An extended cost-effectiveness analysis (ECEA) was conducted to evaluate the distributional impact of improving access to antivenom in urban and rural Lao PDR, where all snakebite envenoming patients with clinical indications of receiving antivenoms could receive antivenom at conventional hospitals, on disease burdens and economic burden. Findings indicated that improving access to antivenom in Lao PDR is essential for reducing the significant burden of snakebite envenoming and addressing health inequities between urban and rural populations. While enhanced availability of antivenom can significantly reduce mortality, it may also impose considerable financial hardships on affected households, particularly in rural areas.

## Introduction

Snakebite envenoming remains a significant cause of preventable mortality. The Global Burden of Disease Study 2019 identified snakebite envenoming as the most fatal neglected tropical disease (NTD), accounting for an estimated 63,400 deaths globally, corresponding to an age-standardized mortality rate (ASMR) of 0.8 per 100,000 population [1]. Antivenom, the only specific treatment for snakebite envenoming, is essential for preventing severe morbidity and death among patients with systemic envenoming and clinical indications, and has been pivotal in reducing the ASMR by 36% since 1990 [1]. However, access to antivenom remains a substantial challenge in many parts of the world, particularly in resource-limited settings [2–7]. Recognizing the critical need to address this global health issue, the World Health Organization (WHO) has designated snakebite envenoming as a highly prioritized NTD and introduced a strategic roadmap to halve its global burden by 2030 [8].

The Lao People’s Democratic Republic (PDR), or Laos, is among the Southeast Asian countries disproportionately affected by snakebite envenoming. Medically important snakes in the Lao PDR include elapids (e.g., kraits and cobras) that cause neurotoxic envenoming, and vipers (e.g., green pit vipers and Malayan pit viper) associated with hematotoxicity and local tissue damage. Epidemiological studies of snakebites in the Lao PDR suggested that viperid bites constitute approximately 70% of reported envenomings, with neurotoxic cases representing a smaller but severe subset. Although reported case fatality rates in Lao PDR are relatively low (up to approximately 3%), these estimates are likely to be underestimated due to limitations in hospital-based mortality data and cultural preferences for dying at home [9–12]. Several systemic challenges, including a lack of reliable national statistics on snakebites and the inadequate supply of antivenoms, impede the management of snakebites in Lao PDR. Antivenoms are mainly sourced from the Queen Saovabha Memorial Institute (QSMI) in Thailand, including monovalent antivenoms for cobra, Malayan krait, Malayan pit viper, and green pit viper, as well as Neuro and Hemato Polyvalent snake antivenins [2]. Antivenoms are available in limited quantities at a few hospitals, with reports indicating that physicians have even had to resort to using expired antivenoms to save their patients’ lives [2,12–15]. Only 4.2% of snakebite envenoming cases in Lao PDR are estimated to receive antivenom [13]. The limited availability has substantially influenced treatment-seeking behavior, with snakebite victims primarily opting for traditional healers over conventional hospitals. This is primarily attributable to the inadequate supply of antivenoms and the substantial financial burden of out-of-pocket (OOP) healthcare expenditures [2,9,11]. Although Lao PDR’s National Health Insurance (NHI) scheme has expanded healthcare coverage, a considerable proportion of total health expenditures is still financed through household OOP payments [16,17]. Consequently, most snakebite victims rely on traditional treatment, which is more affordable than conventional treatment. Victims commonly use unidentified herbal or root-based alcoholic solutions. Cultural beliefs include swallowing the Malayan pit viper’s tail, inserting the bitten limb into an ant hill, or wearing loose ribbons as protective charms. Specific plant-based remedies involve applying pickled bamboo shoots or wrapping *Imperata cylindrica* (Cogon grass) around the limb to prevent venom spread [9,11,18].

Snakebite envenoming is widely recognized as a “disease of poverty,” disproportionately affecting socioeconomically disadvantaged populations [19]. In Lao PDR, the burden of snakebite is concentrated in rural areas, where agricultural activities and proximity to forests increase exposure risk. These rural populations also face greater barriers to accessing care, including the concentration of antivenom supplies in urban hospitals, which exacerbates health inequities between urban and rural communities [2,9,11].

Improving access to antivenom represents a crucial strategy for mitigating the burden of snakebite envenoming. A prior cost-effectiveness analysis (CEA) highlighted the substantial clinical and economic benefits of hypothetically treating all snakebite envenoming cases in Lao PDR with antivenoms, resulting in cost savings of 4,710 United States Dollars (USD), averting 0.06 deaths and 1.5 DALYs per case, thereby constituting a dominant strategy [20]. However, the study did not examine the distributional impacts of antivenom access across urban and rural populations nor evaluate the potential benefits of financial risk protection. While a systematic review has demonstrated the significant financial hardships associated with NTDs [21], the financial hardship of snakebite envenoming in Lao PDR remains underexplored.

To address these gaps, this study employs an extended cost-effectiveness analysis (ECEA) to evaluate the distributional impact of improving access to antivenom on health outcomes and economic burden stratified by urban and rural populations in Lao PDR. ECEA is an equity-informative economic evaluation designed to assess the impacts of public policies on health equity by examining the distribution of health and the financial risks across equity-relevant subpopulations [22]. The findings of our study are intended to inform policymakers in Lao PDR by providing evidence-informed recommendations for developing equitable strategies to address snakebite envenoming, thereby advancing the country’s universal health coverage (UHC) agenda.

## Methods

### Ethical approval

This study did not require ethical approval because it is a modeling study based on secondary data from previously published literature and publicly available sources. No primary data were collected, and no human participants were involved directly. As such, ethical review and approval were not applicable to the scope of this analysis.

### Overall approach

We conducted an ECEA to evaluate the distributional impact of improving access to antivenom in urban and rural areas of Lao PDR. Building on a previous conventional CEA [20], this study additionally examined the distribution of health and financial consequences across equity-relevant subgroups. This ECEA adhered to the methodological framework established by Verguet and colleagues [22] and was reported following the Consolidated Health Economic Evaluation Reporting Standards (CHEERS) 2022 guidelines (S1 Table) [23]. In line with the ECEA framework, we evaluated: (1) health outcomes, including mortality and disability-adjusted life years (DALYs); (2) financial consequences through household out-of-pocket expenditures; and (3) distributional impacts across urban and rural populations in Lao PDR. As household-level income distribution data were unavailable, we did not directly estimate catastrophic health expenditure (CHE), poverty cases averted, or money-metric insurance value. Instead, the percentage of household out-of-pocket (OOP) expenditure relative to monthly household income was used as a proxy indicator of financial hardship and affordability. Annual household income was not used because snakebite envenoming is generally an acute episodic event with high upfront costs during the envenoming episode [19].

Urban and rural areas were defined according to administrative classifications used in national statistics, with urban areas corresponding to provincial capitals and urban districts, and rural areas encompassing populations outside these administrative boundaries [18,24,25]. Approximately 20–30% of snakebite victims have systemic envenoming meeting clinical indications for antivenom treatment [10]. This evaluation compared the “current access” scenario (limited access), where only a small proportion of victims with systemic envenoming meeting clinical indications for antivenom treatment received antivenom treatment in conventional healthcare facilities due to limited supplies, with an alternative hypothetical “full access” scenario, where all victims were assumed to ideally seek conventional treatment, and all snakebite victims with systemic envenoming meeting clinical indications for antivenom treatment were assumed to receive antivenom at the conventional healthcare facilities, rather than universal administration to all snakebite victims.

### Model

A decision model was developed based on the structure and assumptions of a previous CEA [20]. The model was modified to separately estimate the annual number of snakebite victims in urban and rural Lao PDR, accounting for differences in treatment-seeking behavior and healthcare access. Snakebite victims were modeled as seeking care from conventional healthcare facilities (public or private), traditional healers, or receiving no treatment. Antivenom treatment was provided based on the level of access at the conventional healthcare facilities, determined by the number of available antivenoms and the average number of antivenoms used per case (20 and 90 available antivenom treatments in rural and urban Lao PDR, respectively). Patient outcomes included survival, survival with disabilities, or death. This ECEA was conducted from three perspectives: household, healthcare system, and societal.

### Input parameters

Input parameters specific to urban and rural settings were sourced from the prior CEA model [20] and publicly available data, including published literature and government websites [9,10,17,18,24–31]. Specifically, we searched PubMed for published articles on snakebite in the Lao PDR, complemented by Google and Google Scholar searches. Websites of the Ministry of Health of the Lao PDR and the Lao Statistics Bureau were also searched. The search period ended on 30 April 2023. All documents and websites with information on snakebites in the Lao PDR were included, and relevant details were extracted to serve as input parameters for the model. Candidate input parameters were initially identified from published literature, government databases, and official websites by three researchers (C.P., J.C., and N.C.). This process was followed by a virtual group discussion with three researchers (M.M., K.S., and J.B.) who have extensive experience in the clinical management and epidemiology of snakebite envenoming in Lao PDR, to evaluate their relevance and suitability in the Lao PDR context. When data were unavailable, expert opinion was used to estimate plausible parameter values. We validated assumptions through multiple sources and adopted conservative estimates whenever possible. Input parameters are listed in S2 Table.

### Burden of snakebite

We estimated the impact of improving access to antivenom on the disease and economic burdens. Disease burdens included the case fatality rate (CFR), mortality rate, amputations due to snakebite envenoming, and DALYs, comprising years of life lost (YLLs) due to premature death and years lived with disability (YLDs) from snakebite envenoming and amputations. Economic burden was quantified as the costs of snakebites from various perspectives. Financial risk was assessed as the percentage of household OOP expenditure relative to monthly household income, serving as a proxy for financial hardship and affordability.

### Costs

A micro-costing approach was employed to estimate the costs associated with snakebite. Through group discussion, three local clinicians (M.M., K.S., and J.B.) estimated the average resource utilization for managing snakebite patients in the context of Lao PDR (S3-S7 Tables). Direct medical costs included hospitalization, inpatient services, laboratory tests, tetanus toxoid administration, wound care, antivenom treatment, management of adverse reactions, amputations, and traditional treatment. Direct non-medical costs included transportation and additional food expenses for patients and their caregivers. Transportation costs were assumed to include only cars and motorcycles. Inter-facility transportation, medical evacuation, and ambulance cars were not captured in the costing framework. Indirect costs were calculated as productivity losses due to premature death and the productivity losses by patients and caregivers during treatment episodes. Local stakeholders indicated that, on average, five family members assisted in caregiving. All costs were reported in 2023 USD, with a conversion rate of 14,035.23 Laotian Kip (LAK) [32].

Household perspective included OOP expenditures on snakebite medical treatment, transportation, and food for victims and family members. The healthcare system perspective included direct medical costs for snakebites. The societal perspective included household and health system costs and indirect costs.

### Base-case analysis

A base-case analysis was performed to estimate the health equity impact of improving access to antivenom, defined as differences in the burden of snakebite between urban and rural populations. Equity improvements were achieved if these differences decreased with improved access. Primary analysis was performed using the household perspective. Secondary analyses were performed using the healthcare system and societal perspectives. Additionally, we analyzed the cost distribution associated with snakebite in urban and rural areas to identify key drivers of societal and household OOP expenditures.

### Sensitivity analyses

Several sensitivity analyses were conducted to assess uncertainties and explore the robustness of the results. Probabilistic sensitivity analysis (PSA) was performed using a Monte Carlo simulation with 1,000 iterations randomly sampled from a distribution of input parameters from distributions such as gamma (e.g., length of hospital stay) and beta (e.g., probabilities of adverse reactions, survival outcomes, and disability weights) (S2 Table). The 95% credible interval (95% CrI) for health equity impacts was estimated from the PSA to reflect the range of uncertainty in the model findings.

As Laotians cover 41.8% of their total health expenditure through OOP expenses [17], scenario analyses were conducted to examine the effects of changes in coverage of antivenom treatment and direct medical costs on OOP expenditures as a percentage of monthly household income. These analyses assumed coverage of medical care costs ranging from 0 to 100%, serving as a proxy for improving healthcare affordability under a fully implemented UHC system in Lao PDR.

Further scenario analyses were performed to evaluate the impact of no transportation expenditures, and only one caregiver assisted the victim. As some patients in rural areas might need to travel up to 20 km to access care [33], these analyses simulated the effect of improved geographic accessibility with increased availability of healthcare facilities closer to patient residences, resulting in lower costs for transportation and caregiving.

Two-way sensitivity analyses were performed to explore the combined effects of improved access to antivenom and changes in treatment-seeking behaviors. Additionally, two-way analyses evaluated the interaction between increased coverage of medical expenditures (from 58.2% to 100%) and reductions in transportation expenditures (from 0% to 100%) on household OOP expenditures in urban and rural areas. These analyses aimed to elucidate the interplay between geographical accessibility and healthcare affordability.

## Results

### Estimated burden of snakebite in urban and rural Lao PDR

The estimated epidemiological, economic, and disease burdens of snakebites in urban and rural Lao PDR under current and full access scenarios are shown in Table 1. Annually, there were an estimated 1,506 snakebite victims in urban areas and 13,553 in rural areas.

**Table 1:** Estimated burden of snakebite in urban and rural Lao PDR.

	Current access		Full access^*^	
	Urban	Rural	Urban	Rural
**Epidemiological burden**				
**1. Total snakebite patient, n**	**1,506**	**13,553**	**1,506**	**13,553**
1.1 Incidence rate per 100,000 population	53	288	53	288
**2. Snakebite envenoming, n**	**301**	**2,963**	**301**	**2,963**
2.1 Conventional treatment, n	241	469	301	2,963
2.1.1 Received antivenom, n	20	90	301	2,963
2.1.3 Not receive antivenom, n	221	379	0^*^	0^*^
2.2 Traditional treatment, n	60	2,494	0^*^	0^*^
2.3 Receive antivenom, %	6.6%	3.0%	100%^*^	100%^*^
**3. Snakebite without systemic envenoming, n**	**1,205**	**10,590**	**1,205**	**10,590**
3.1 Conventional treatment, n	964	887	1,205	10,590
3.2 Traditional treatment, n	241	9,704	0^*^	0^*^
**4. Death from snakebite envenoming, n**	**10**	**147**	**7**	**69**
4.1 Case fatality rate of snakebite envenoming	3.3%	5.0%	2.3%	2.3%
4.2 Mortality rate per 100,000 population	0.35	3.14	0.25	1.47
**5. Amputation from snakebite envenoming, n**	**20**	**244**	**21**	**250**
5.1 Disability rate per 100,000 population	0.43	5.18	0.44	5.33
**Economic burden**				
**1. All snakebite**				
1.1 Societal perspective, USD	744,077	7,979,430	777,276	7,888,453
1.1.1 per person, USD	494	589	516	582
1.2 Healthcare system perspective, USD	109,396	240,199	262,950	3,034,663
1.2.1 per person, USD	73	18	175	224
1.3 Household perspective, USD	142,306	837,339	210,469	2,007,713
1.3.1 per person, USD	94	62	140	148
1.3.2%OOP/monthly household income (all victims)	38%	39%	56%	95%
1.3.3%OOP/monthly household income (hospital only)	42%	87%	56%	95%
1.3.4%OOP/monthly household income (traditional healers)	22%	34%	–	–
**2. Snakebite with systemic envenoming**				
2.1 Societal perspective, USD	603,613	7,227,457	621,220	6,420,070
2.1.1 per person, USD	2,004	2,439	2,063	2,167
2.2 Healthcare system perspective, USD	61,465	187,292	203,037	2,402,669
2.2.1 per person, USD	204	63	674	811
2.3 Household perspective, USD	64,455	257,826	129,289	1,294,594
2.3.1 per person, USD	214	87	429	437
2.3.2%OOP/monthly household income (all victims)	86%	56%	173%	279%
2.3.3%OOP/monthly household income (hospital only)	102%	169%	173%	279%
2.3.4%OOP/monthly household income (traditional healers)	22%	34%	–	–
**3. Snakebite without systemic envenoming**				
3.1 Societal perspective, USD	140,464	751,973	156,056	1,468,383
3.1.1 per person, USD	117	71	130	139
3.2 Healthcare system perspective, USD	47,930	52,907	59,913	631,994
3.2.1 per person, USD	40	5	50	60
3.3 Household perspective, USD	77,851	579,512	81,180	713,119
3.3.1 per person, USD	65	55	67	67
3.3.2%OOP/monthly household income (all victims)	26%	35%	27%	43%
3.3.3%OOP/monthly household income (hospital only)	27%	43%	27%	43%
3.3.4%OOP/monthly household income (traditional healers)	22%	34%	–	–
**Disease burden**				
**1. DALYs lost per 100,000 population**				
1.1 Snakebite envenoming	8.57	76.36	6.04	35.79
1.2 Snakebite without systemic envenoming	0.001	0.004	0.001	0.004
1.3 Amputation	0.28	2.61	0.29	2.68
1.4 Total	8.86	78.97	6.33	38.47

Note: * – It was assumed that all snakebite victims sought conventional treatment and all victims with snakebite envenoming received antivenom treatment; 1 United States Dollar = 14,035.23 Laotian Kip. Differences were calculated using unrounded model outputs. Values presented are rounded for readability; therefore, arithmetic differences based on displayed values may not exactly match the reported differences. **Abbreviations:** DALYs – Disability-adjusted life years; OOP – Out-of-pocket; USD – United States Dollar.

In urban areas, 301 snakebite envenoming cases were estimated, with 20 patients (6.6%) receiving antivenom treatment. This resulted in 10 deaths, corresponding to a CFR of 3.3%. The total societal cost of snakebites in urban areas was estimated at 744,077 USD, equivalent to 494 USD per victim. The disease burden was 8.86 DALYs lost per 100,000 population. Household OOP expenditures for snakebite treatment were estimated at 38% of the monthly household income for all victims. Victims with systemic envenoming treated in hospitals faced the highest financial risk, with OOP expenditures reaching 102% of monthly household income.

Rural areas were estimated to have 2,963 snakebite envenoming cases, with only 90 patients (3.0%) receiving antivenom treatment. These cases resulted in 147 deaths, corresponding to a CFR of 5.0%. The total societal cost of snakebites in rural areas was significantly higher, at 7,974,430 USD, or 589 USD per victim. The disease burden was 78.97 DALYs lost per 100,000 population. Household OOP expenditures for snakebite treatment were estimated at 39% of monthly household income for all victims. Victims with systemic envenoming treated in hospitals also faced the highest financial risk, with OOP expenditures reaching 169% of monthly household income.

### Health equity impact of improving access to antivenom in urban and rural Lao PDR

Under the current access scenario, snakebite victims in rural areas experienced worse epidemiological outcomes and higher economic burdens compared to urban areas. Rural areas had higher mortality rates (+2.79 deaths per 100,000 population; 95% CrI + 0.40 to +4.29), DALYs lost (+70.11 DALYs per 100,000 population; 95% CrI + 12.04 to +105.47) and household economic burden (+1% OOP expenditures per monthly household income, 95% CrI -4% to +6%), than urban areas. These differences were associated with considerable uncertainty, as reflected in the wide credible intervals.

**Fig 1:**
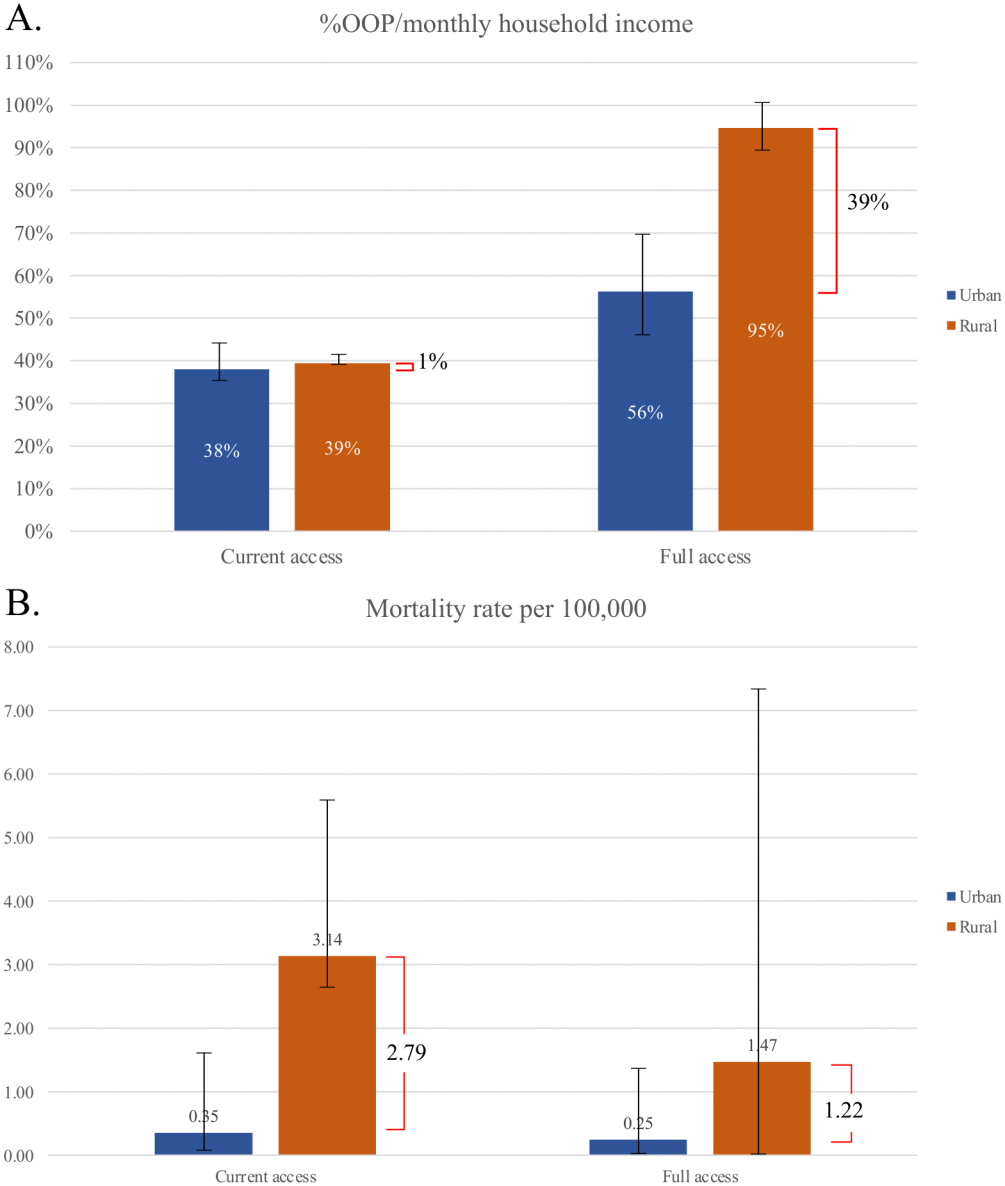
Health equity impact of improving access to antivenom in financial risk and mortality of snakebite in urban and rural Lao PDR. Full access was assumed that all snakebite victims sought conventional treatment and all victims with snakebite envenoming received antivenom treatment. Red lines indicate differences (health equality impact) between urban and rural areas. Black lines indicate a 95% credible interval from 1,000 probabilistic sensitivity analysis simulations. Differences were calculated using unrounded model outputs. Values presented are rounded for readability; therefore, arithmetic differences based on displayed values may not exactly match the reported differences. **Abbreviation:** OOP – Out-of-pocket.

Improving access to antivenom reduced health inequities in deaths and DALYs lost between urban and rural areas. However, it exacerbated health inequities in the household economic burden. In the full access scenario, while rural areas still showed worse epidemiological outcomes than urban areas, the health inequities in mortality and DALYs lost were reduced. Rural areas exhibited higher mortality rates (+1.22 deaths per 100,000 population; 95% CrI -0.95 to +7.09) and greater DALYs lost (+32.14 DALYs per 100,000 population; 95% CrI -24.23 to +193.16) compared to urban areas. The household economic burden in rural areas increased significantly compared to urban areas (95% vs. 56% of OOP expenditures per monthly household income), with 39% higher OOP expenditures per monthly household income (95% CrI + 23% to +52%). Wide credible intervals indicated considerable uncertainty about these differences. These findings are presented in Table 2 and Fig 1.

**Table 2:** Health equity impact of improving access to antivenom in urban and rural Lao PDR.

	Current access			Full access^*^		
	Urban	Rural	Difference (Rural-Urban)	Urban	Rural	Difference (Rural-Urban)
%OOP/monthly household income (all victims)	38% (34 to 44)	39% (39 to 41)	+1% (-4 to +6)	56% (46 to 70)	95% (88 to 101)	+39% (+23 to +52)
%OOP/monthly household income (hospital only)	42% (36 to 49)	87% (77 to 99)	+44% (+33 to +57)	56% (46 to 70)	95% (88 to 101)	+39% (+23 to +52)
Case fatality rate of snakebite envenoming	3.3% (0.8 to 14.7)	5.0% (4.1 to 8.7)	+1.7% (-11.4 to +5.3)	2.3% (0 to 12.1)	2.3% (0.0 to 11.7)	0.0% (-10.8 to +10.8)
Mortality rate per 100,000 population	0.35 (0.10 to 1.55)	3.14 (2.64 to 5.48)	+2.79 (+0.40 to +4.29)	0.25 (0 to 1.32)	1.47 (0 to 7.35)	+1.22 (-0.95 to +7.09)
Total DALYs lost per 100,000 population	8.86 (2.67 to 38.17)	78.97 (65.80 to 136.33)	+70.11 (+12.04 to +105.47)	6.33 (0.11 to 32.30)	38.47 (1.64 to 198.10)	+32.14 (-24.23 to +193.16)

Note: * – It was assumed that all snakebite victims sought conventional treatment and all victims with snakebite envenoming received antivenom treatment; Numbers in parentheses show the range of 95% credible interval from 1,000 probabilistic sensitivity analysis simulations. Differences were calculated using unrounded model outputs. Values presented are rounded for readability; therefore, arithmetic differences based on displayed values may not exactly match the reported differences. **Abbreviations:** DALYs – Disability-adjusted life years; OOP – Out-of-pocket.

**Fig 2:**
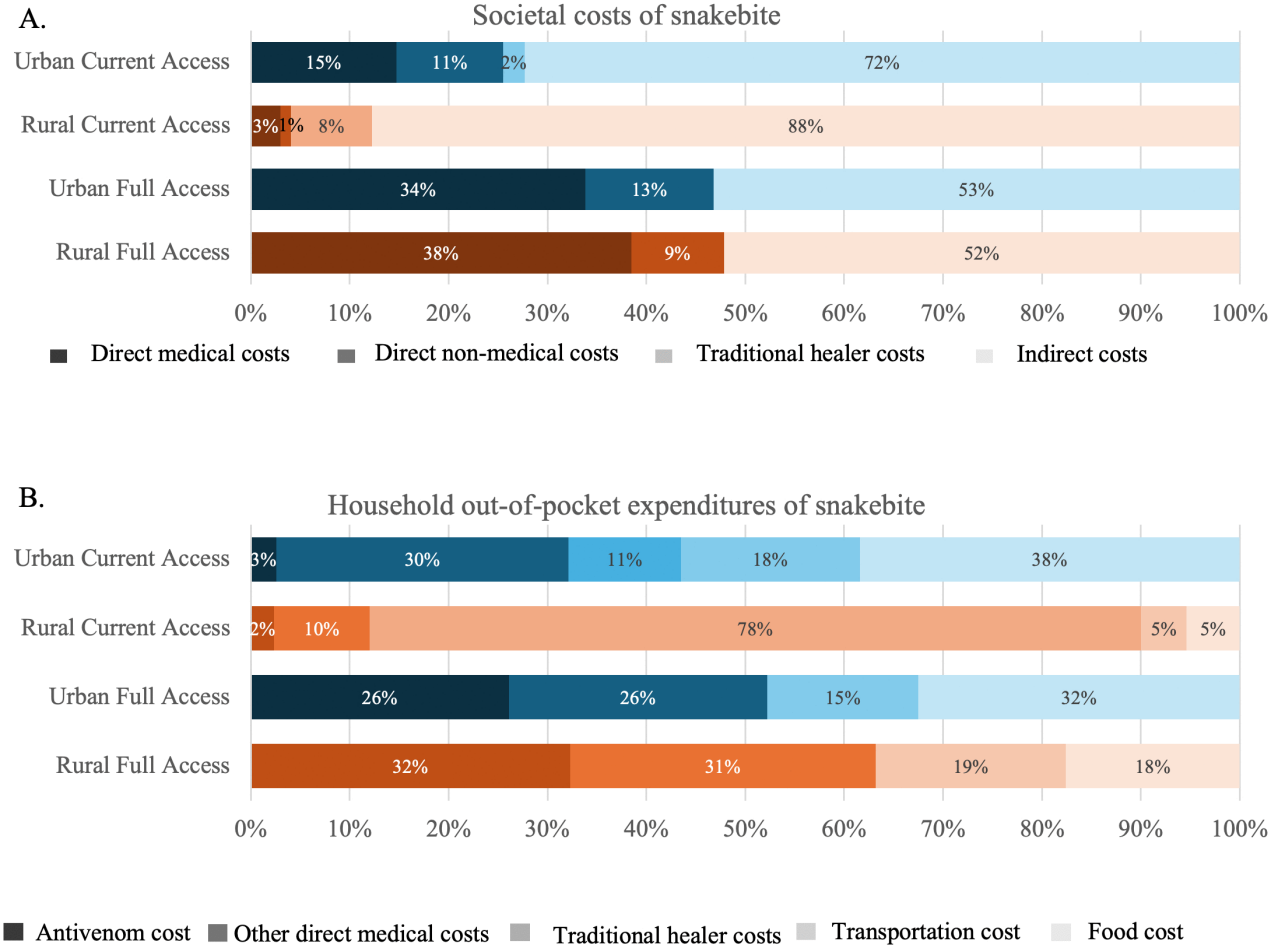
Distribution of costs of snakebite in urban and rural Lao PDR. Full access was assumed that all snakebite victims sought conventional treatment and all victims with snakebite envenoming received antivenom treatment. Values presented are rounded for readability.

The distribution of snakebite costs in urban and rural Lao PDR is shown in Fig 2. Indirect costs constituted most of the societal costs of snakebites, accounting for more than half of the total of each scenario across urban and rural areas (52–88%). Full access to antivenom led to a marked increase in the proportion of direct medical costs in both areas. Direct non-medical costs showed a substantial rise only in rural areas under improved access conditions. An analysis of the distribution of household OOP expenditures for snakebites demonstrated that food (38%) and hospitalization costs (18%) were the cost drivers in urban areas. Cost drivers in rural areas were mostly traditional healer costs (78%). Improving access to antivenom increased the relative share of direct medical costs in urban and rural areas.

### Sensitivity analyses

**Fig 3:**
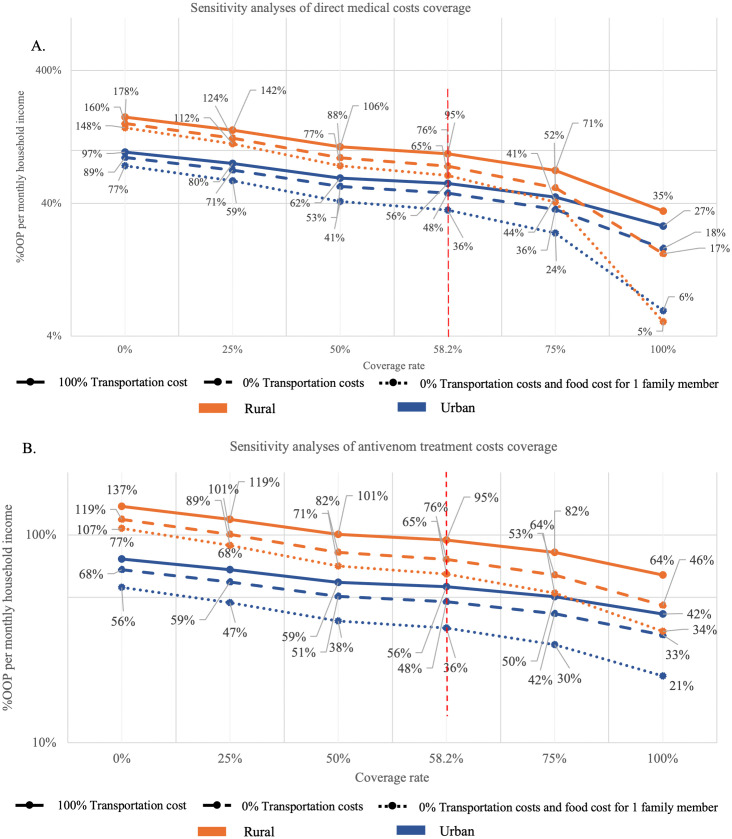
Scenario analyses of the impact of coverage of direct medical costs, transportation cost, and antivenom on the household out-of-pocket expenditure in full access to antivenom in urban and rural Lao PDR. Full access was assumed that all snakebite victims sought conventional treatment and all victims with snakebite envenoming received antivenom treatment. Scenario analyses were performed to see the impact of varying the coverage of direct medical costs and antivenom on the percentage of OOP expenditures per household monthly income. Red dashed lines indicate the current coverage of medical services at 58.2%. The residual household OOP expenditures resulted from non-reimbursable direct medical costs and OOP direct non-medical costs. Further analyses were performed by reducing the transportation costs to none (Solid lines vs Dashed lines), indicating the residual household OOP expenditures on food. Lastly, additional scenario analyses were performed by assuming only one family member assisting the patient in caregiving during hospitalization, as shown in the dotted lines. Differences were calculated using unrounded model outputs. Values presented are rounded for readability; therefore, arithmetic differences based on displayed values may not exactly match the reported differences. **Abbreviation:** OOP – Out-of-pocket.

Scenario analyses examining the impact of coverage of direct medical costs and antivenom treatment on household OOP expenditures under the full access scenario in urban and rural Lao PDR are presented in Fig 3. Full coverage of direct medical costs provided an even greater reduction in health inequities, with urban costs decreasing from 56% to 27% and rural costs from 95% to 35%. Increasing the coverage of antivenom treatment to 100% significantly reduced health inequities in %OOP expenditures per monthly household income, lowering costs from 56% to 42% in urban areas and 95% to 64% in rural areas.

Zero transportation costs on top of full coverage of direct medical costs reduced health inequities in %OOP expenditures per monthly household income, lowering costs from 27% to 18% in urban areas and 35% to 17% in rural areas. Additional scenario analyses assuming only one family member assisting the patient in caregiving during hospitalization further reduced health inequities in %OOP expenditures per monthly household income, lowering costs from 18% to 6% in urban areas and 17% to 5% in rural areas.

Two-way sensitivity analyses, presented in S8 Table, evaluated the combined impact of access to antivenom and treatment-seeking behavior on %OOP expenditures per monthly household income in urban and rural Lao PDR. The results demonstrated that improving access to antivenom and shifting treatment-seeking behavior toward conventional healthcare facilities substantially influenced the household economic burden. Additional two-way sensitivity analyses, presented in S9 Table, evaluated the impact of transportation costs and coverage of direct medical costs on %OOP expenditures per monthly household income. Improving the coverage of direct medical costs and reducing transportation costs reduced the financial impacts on households in both areas.

## Discussion

Our research highlighted significant health inequities between urban and rural communities in Lao PDR concerning access to antivenom. This ECEA adjusted the model from the previous study, which considered all victims without differentiating between rural and urban areas. As a result, the previous model, along with its input parameters and assumptions, was modified to estimate the new findings presented in this ECEA, demonstrating the impact of improved access between rural and urban areas on the burden of snakebite for society, the health system, and households in Lao PDR. Snakebite envenoming remains an important public health challenge in low-resource settings, and the findings from this ECEA underscored both the obstacles and opportunities associated with improving antivenom accessibility. The estimates revealed pronounced health inequities in snakebite-related outcomes, with rural areas experiencing higher CFR and DALYs lost compared to urban regions. These health inequities are likely driven by insufficient healthcare infrastructure, geographic barriers, and the limited availability of antivenom in rural settings, which collectively exacerbate the vulnerability of rural populations [2,9–12]. Our study provides a quantitative assessment of these health inequities through the lens of financial risk associated with snakebite envenoming, particularly highlighting the economic vulnerability of rural households due to limited healthcare coverage and restricted access to essential services.

Our analysis further demonstrated the significant financial hardships households affected by snakebites face. Although improving access to antivenom effectively reduced the epidemiological burden in urban and rural areas, it paradoxically increased household financial hardships, particularly in rural settings. Snakebite treatment costs and expenses for food and transportation for patients and family members drove this increase in OOP expenditures. These costs were disproportionately higher in rural areas, with less developed healthcare infrastructure. As household incomes in rural areas are substantially lower than urban areas, smaller OOP payments represented a larger share of monthly household income, as reflected in the %OOP per monthly household income. Our estimates of the full access indicate that household OOP expenditures for snakebite reached 56% and 95% of monthly household income in urban and rural areas, respectively. Because a single snakebite episode consumes a disproportionate share of monthly resources, these figures illustrate the severe liquidity and affordability shock that rural households would face. Such costs pose a significant risk of driving households into, or further into, poverty, particularly in rural areas where the need for treatment is greatest.

While improving access to antivenom reduced the epidemiological burden, the two-way sensitivity analyses revealed that a shift in treatment-seeking behavior combined with enhanced access to healthcare significantly increased household OOP expenditures. Sensitivity analyses demonstrated that full coverage of snakebite treatment could mitigate financial risks and reduce health inequities between urban and rural areas. However, the coverage was insufficient to avoid the financial hardship as the residual household OOP expenditures incurred by transportation and food. Our scenario analyses suggest that full coverage of antivenom and snakebite treatment, along with easily accessible healthcare facilities—specifically, zero transportation costs and one family member providing care—could ultimately reduce household OOP expenditures to 6% in urban areas and 5% in rural areas. These findings align with the prior systematic review of the financial hardship among patients suffering from NTDs, which demonstrated that even when drugs for treating NTDs were supplied at no cost, expenditures for supportive care, medical procedures, transportation, and food remained substantial, potentially leading to a severe financial constraint on these families [34].

Therefore, our findings underscore the critical insight that improving antivenom availability alone is insufficient to achieve health equity. Comprehensive strategies that address financial and geographical barriers are required to ensure equitable access to healthcare and alleviate the disproportionate burden rural communities face.

### Limitations

This ECEA should be interpreted with the following limitations. Firstly, input parameters were derived from a handful of published observational studies of snakebites in the Lao PDR [9–12]. In addition, when data were unavailable, we relied on expert opinion obtained through group discussions with researchers and clinicians experienced in the clinical management and epidemiology of snakebite envenoming in Lao PDR. However, we did not use formal expert elicitation methods, such as structured interviews or Delphi techniques, which may limit the reproducibility and methodological rigor of the parameter estimation process. To address the inherent uncertainty in these input parameters, we validated our assumptions using multiple sources and adopted conservative estimates where possible. Secondly, while snakebite envenoming can result in multiple physical and mental complications and disabilities, such as secondary infections, surgical complications, acute kidney injuries, post-traumatic stress disorder, or anxiety [35,36], our model included only amputations, as they are the only disability of snakebite, for which we had sufficient evidence to incorporate into the model [10]. Severe anaphylactic shock due to antivenom was also not modeled separately. This simplification may underestimate some harms and costs associated with antivenom treatment. However, severe fatal reactions to antivenom are expected to be uncommon based on currently available evidence [30]. Thus, we adopted a conservative modeling approach and acknowledge that excluding additional complications and long-term sequelae may underestimate the overall burden of snakebite and the potential benefits of improving access to antivenom in Lao PDR. Incorporating these complications would likely increase the estimated burden of snakebite and OOP expenditures, particularly in rural populations, and may further widen the observed urban–rural inequities. This constraint underscores the need for further research to capture the broader spectrum of snakebite-related complications, sequelae, and disabilities in Lao PDR. Thirdly, we assumed that antivenom administration improves outcomes across all snakebite cases. While clinical outcomes vary by snake type and time to treatment, our model applies an average effectiveness across cases due to the lack of species-specific real-world data in Lao PDR. We acknowledge this may overestimate the impact in delayed cases or for envenomings where antivenom is less effective. Fourthly, mortality estimates in this study were primarily derived from hospital-based data and may not fully capture deaths occurring outside the formal healthcare system. In rural areas of Lao PDR, some snakebite victims may never reach healthcare facilities because of geographical barriers, delayed care-seeking, or reliance on traditional healers. In addition, some severely ill patients may be discharged home due to cultural preferences for dying at home, resulting in deaths that are not captured in hospital mortality statistics. Therefore, the mortality burden of snakebite envenoming in the Lao PDR may be underestimated in this analysis [9–12]. Lastly, the analysis did not incorporate household-level income distributions and therefore could not estimate the incidence of CHE. The %OOP per monthly household income should be interpreted as a proxy of financial risk protection.

Despite these limitations, modeling studies remain important for quantifying disease burden and informing mitigation strategies. Although models have been developed to estimate the burden of snakebites in various global settings [37], comprehensive studies are still needed to refine input parameters and enhance accuracy. While data availability is often considered a prerequisite for modeling, in some cases, modeling may serve as a starting point to guide data collection and research efforts. This approach could be particularly relevant for NTDs, where data gaps persist and may hinder effective policy development. Combining modeling with empirical research may help improve the evidence base for addressing the snakebite burden, particularly in low- and middle-income countries such as Lao PDR.

### Policy implications

The findings from our ECEA highlight the urgent need for equity-focused strategies to address the health inequities in snakebite envenoming outcomes between urban and rural communities in Lao PDR. Our model incorporates expert-informed differential mortality by geography, reflecting real-world barriers to effective treatment in rural settings, and highlights the importance of integrated health system responses. However, antivenom access alone is unlikely to achieve optimal outcomes without concurrent investments in timely access to care, supportive treatment (e.g., ventilation, transfusions), health worker training, and community education. Policymakers should view antivenom access as a foundational but not a standalone intervention. Antivenom is crucial for treating systemic envenoming, and timely use prevents severe complications and death. Due to resource limits in low-income settings such as Lao PDR, antivenom should be targeted, not universal. Improving clinical triage, following guidelines, and early detection are key to using limited supplies efficiently and equitably. Training healthcare workers to recognize indications for antivenom, alongside improvements in referral pathways and supportive care capacity, can maximize the health gains achievable from existing antivenom resources while minimizing unnecessary use.

Furthermore, improving the accessibility, availability, and affordability of snakebite treatment is needed. While the NHI scheme has successfully expanded coverage in Lao PDR [16], increasing coverage of antivenom and associated medical costs is essential to alleviate the financial burden on households, particularly in rural areas. Transportation subsidies within the NHI framework can further reduce economic barriers to access to care. These measures would address the high OOP expenditures pushing many rural households into catastrophic health spending. However, increasing coverage and introducing subsidies require additional financial resources, which can be challenging for a low-resource country like Lao PDR. The government could explore international funding opportunities and regional collaborations to overcome this. Partnering with global health organizations, such as WHO and international donors, could provide financial and technical support. Additionally, regional pooled procurement systems, mainly through ASEAN partnerships [2], could help reduce antivenom costs, making increased coverage and subsidies more feasible. Advocacy efforts emphasizing the economic and health equity benefits of these strategies can help secure commitments from government policymakers and donors.

Efforts should focus on shifting treatment-seeking behaviors from traditional to conventional care. Targeted awareness campaigns and community engagement are critical to encouraging timely medical treatment. Training healthcare workers in rural facilities to manage snakebite envenoming effectively is essential for building trust in healthcare systems and ensuring prompt and effective care. Ensuring access to antivenom also requires strengthening procurement and supply chain mechanisms. Policymakers could explore regional pooled procurement and international collaborations to secure affordable, high-quality antivenom supplies. Improving inventory management systems at local health facilities would further ensure that antivenom is available where it is needed most. Establishing a national snakebite registry would improve data collection, enabling policymakers to monitor snakebite incidence, treatment outcomes, and associated economic impacts. In parallel, prevention strategies remain critical for reducing the incidence of snakebite, particularly in rural and agricultural settings. Environmental and occupational risk-reduction measures, such as community education on snake avoidance, the use of protective equipment during agricultural work, improved housing conditions, and targeted interventions in high-risk areas, should be integrated with healthcare system strengthening efforts. These policy implications, aligned with existing health system efforts and international best practices [2,8,16], can significantly reduce the health and economic burden of snakebite envenoming.

## Conclusion

Improving access to antivenom in Lao PDR is essential for reducing the significant burden of snakebite envenoming and addressing health inequities between urban and rural populations. While enhanced availability of antivenom can significantly reduce mortality, it may also impose considerable financial hardships on affected households, particularly in rural areas. These findings highlight the need for targeted health policies that ensure equitable access to snakebite treatment while providing financial protection to mitigate the economic burden. Such policy initiatives should focus on reducing OOP expenditures through increased coverage, expanded healthcare coverage, and improved geographical accessibility to healthcare facilities. These strategies will enable both urban and rural communities to access life-saving treatments, aligning with Lao PDR’s UHC objectives and the WHO’s goal of reducing the burden of NTDs.

## Supporting information

S1 Table CHEERS 2022. (DOCX)

S2 Table Input parameters. (DOCX)

S3 Table Micro-costing of Hospitalization costs for snakebite victims with systemic envenoming. (DOCX)

S4 Table Micro-costing of Hospitalization costs for victims without snakebite envenoming. (DOCX)

S5 Table Micro-costing of antivenom treatment costs. (DOCX)

S6 Table Micro-costing of adverse reaction management costs. (DOCX)

S7 Table Micro-costing of other costs. (DOCX)

S8 Table Two-way sensitivity analyses of the impact of access to antivenom and treatment-seeking behavior on out-of-pocket expenditures for snakebite per monthly household income in urban and rural Lao PDR. (DOCX)

S9 Table Two-way sensitivity analyses of the impact of transportation cost and coverage of direct medical costs on out-of-pocket expenditures for snakebite per monthly household income in urban and rural Lao PDR. (DOCX)
