# Supplementary material for: Estimating the distributional impact of improving access to snake antivenom in urban and rural Lao People’s Democratic Republic: An extended cost-effectiveness analysis
Source: PLoS Negl Trop Dis. 2026 Jun 4;20(6):e0014420. doi: 10.1371/journal.pntd.0014420 (PMC13268137; doi:10.1371/journal.pntd.0014420)
Supplement: S1 Table — (DOCX) [file pntd.0014420.s001.docx]

**S1 Table: CHEERS 2022**

| **Topic** | **No.** | **Item** | **Location where item is reported** | **Page No.** |
| --- | --- | --- | --- | --- |
| **Title** | 1 | Identify the study as an economic evaluation and specify the interventions being compared. | TITLE | 1 |
| **Abstract** | 2 | Provide a structured summary that highlights context, key methods, results, and alternative analyses. | ABSTRACT | 4 |
| **Introduction** | | | |  |
| **Background and objectives** | 3 | Give the context for the study, the study question, and its practical relevance for decision making in policy or practice. | INTRODUCTION | 5-7 |
| **Methods** | | | |  |
| **Health economic analysis plan** | 4 | Indicate whether a health economic analysis plan was developed and where available. | Not performed | - |
| **Study population** | 5 | Describe characteristics of the study population (such as age range, demographics, socioeconomic, or clinical characteristics). | METHODS; Model | 8 |
| **Setting and location** | 6 | Provide relevant contextual information that may influence findings. | METHODS; Overall approach | 8 |
| **Comparators** | 7 | Describe the interventions or strategies being compared and why chosen. | METHODS; Overall approach | 8 |
| **Perspective** | 8 | State the perspective(s) adopted by the study and why chosen. | METHODS; Model | 8 |
| **Time horizon** | 9 | State the time horizon for the study and why appropriate. | METHODS; Model | 8 |
| **Discount rate** | 10 | Report the discount rate(s) and reason chosen. | Table S2 | Supplementary material,  Page 5 |
| **Selection of outcomes** | 11 | Describe what outcomes were used as the measure(s) of benefit(s) and harm(s). | METHODS; Burden of snakebite | 9 |
| **Measurement of outcomes** | 12 | Describe how outcomes used to capture benefit(s) and harm(s) were measured. | METHODS; Burden of snakebite | 9 |
| **Valuation of outcomes** | 13 | Describe the population and methods used to measure and value outcomes. | METHODS; Burden of snakebite | 9 |
| **Measurement and valuation of resources and costs** | 14 | Describe how costs were valued. | METHODS; Costs | 9 |
| **Currency, price date, and conversion** | 15 | Report the dates of the estimated resource quantities and unit costs, plus the currency and year of conversion. | METHODS; Costs | 9 |
| **Rationale and description of model** | 16 | If modelling is used, describe in detail and why used. Report if the model is publicly available and where it can be accessed. | METHODS; Model | 8 |
| **Analytics and assumptions** | 17 | Describe any methods for analyzing or statistically transforming data, any extrapolation methods, and approaches for validating any model used. | METHODS; Base-case analysis | 10 |
| **Characterising heterogeneity** | 18 | Describe any methods used for estimating how the results of the study vary for subgroups. | N/A | - |
| **Characterising distributional effects** | 19 | Describe how impacts are distributed across different individuals or adjustments made to reflect priority populations. | METHODS; Base-case analysis | 10 |
| **Characterising uncertainty** | 20 | Describe methods to characterize any sources of uncertainty in the analysis. | METHODS; Sensitivity analyses | 10 |
| **Approach to engagement with patients and others affected by the study** | 21 | Describe any approaches to engage patients or service recipients, the general public, communities, or stakeholders (such as clinicians or payers) in the design of the study. | METHODS; Input parameters | 8 |
| **Results** | | | |  |
| **Study parameters** | 22 | Report all analytic inputs (such as values, ranges, references) including uncertainty or distributional assumptions. | Tables S2-S8 | Supplementary material,  Page 5-11 |
| **Summary of main results** | 23 | Report the mean values for the main categories of costs and outcomes of interest and summarize them in the most appropriate overall measure. | Table 1, Figures 1-2 | 26, 28, 29 |
| **Effect of uncertainty** | 24 | Describe how uncertainty about analytic judgments, inputs, or projections affect findings. Report the effect of choice of discount rate and time horizon, if applicable. | Table S8, Figure 3 | 30,  Supplementary material,  Page 11 |
| **Effect of engagement with patients and others affected by the study** | 25 | Report on any difference patient/service recipient, general public, community, or stakeholder involvement made to the approach or findings of the study | Not performed | - |
| **Discussion** | | | |  |
| **Study findings, limitations, generalizability, and current knowledge** | 26 | Report key findings, limitations, ethical or equity considerations not captured, and how these could affect patients, policy, or practice. | Discussion | 17-19 |
| **Other relevant information** | | | |  |
| **Source of funding** | 27 | Describe how the study was funded and any role of the funder in the identification, design, conduct, and reporting of the analysis | Funding/Support | 21 |
| **Conflicts of interest** | 28 | Report authors conflicts of interest according to journal or International Committee of Medical Journal Editors requirements. | Declaration of interests | 21 |

Consolidated Health Economic Evaluation Reporting Standards (CHEERS) 2022 statement^23^
