# Supplementary material for: Estimating the distributional impact of improving access to snake antivenom in urban and rural Lao People’s Democratic Republic: An extended cost-effectiveness analysis
Source: PLoS Negl Trop Dis. 2026 Jun 4;20(6):e0014420. doi: 10.1371/journal.pntd.0014420 (PMC13268137; doi:10.1371/journal.pntd.0014420)
Supplement: S2 Table — (DOCX) [file pntd.0014420.s002.docx]

**S2 Table: Input Parameters for 2023 Estimates of Snakebite in Urban And Rural Lao PDR**

| Parameters (Distribution) | Urban areas | | Rural areas | |
| --- | --- | --- | --- | --- |
|  | **Value** | **Sources** | **Value** | **Sources** |
| Total population, people | 2,830,480 | World Bank, 2023^25^ | 4,698,995 | World Bank, 2023^25^ |
| Proportion of snakebite victims with incidence of snakebite 200 per 100,000 population based on expert opinion (Fixed) | 10% | Expert opinion | 90% | Expert opinion |
| Probability of snakebite victims seeking conventional treatment only (Fixed) | 50% | Expert opinion | 0% | Expert opinion |
| Probability of snakebite victims seeking traditional treatment only (Fixed) | 20% | Expert opinion | 90% | Expert opinion |
| Probability of snakebite victims firstly seeking traditional treatment then switching to conventional treatment (Fixed) | 30% | Expert opinion | 10% | Expert opinion |
| Probability of systemic envenoming indicated for antivenom treatment for victims seeking conventional treatment (Fixed) | 20% | Expert opinion,  Lower boundary from Vongphoumy 2016^10^ | 35% | Expert opinion,  Upper boundary from Vongphoumy 2016^10^ |
| Probability of systemic envenoming indicated for antivenom treatment for victims seeking traditional treatment (Fixed) | 20% | Expert opinion,  Lower boundary from Vongphoumy 2016^10^ | 20% | Expert opinion,  Lower boundary from Vongphoumy 2016^10^ |
| Number of available antivenoms (Fixed) | 100 | Expert’s information | 450 | Expert’s information |
| Number of available antivenom treatments (5 vials per case based on expert opinion) (Fixed) | 20 | Expert’s information | 90 | Expert’s information |
| Probability of adverse reaction following antivenom treatment (Beta) | 23%  (38-69%) | Expert opinion,  Sriapha 2022^30^ | 23%  (38-69%) | Expert opinion,  Sriapha 2022^30^ |
| Probability of death in snakebite victims without systemic envenoming (Fixed) | 0% | Assumption | 0% | Assumption |
| Probability of death in systemic envenoming treated with antivenom (Beta) | 2%  (1-16%) | Expert opinion, Vongphoumy 2016^10^ | 2%  (1-16%) | Expert opinion,  Vongphoumy 2016^10^ |
| Relative risk of death when antivenoms are not available (Fixed) | 1.26 | Expert opinion,  Lower boundary from Habib 2013^26^ | 2.33 | Expert opinion, Habib 2013^26^ |
| Probability of death in systemic envenoming treated in hospital without antivenom (Fixed) | 3% | Expert opinion, Vongphoumy 2016^10^, Lower boundary from Habib 2013^26^ | 5% | Expert opinion,  Vongphoumy 2016^10^,  Habib 2013^26^ |
| Probability of death in systemic envenoming not treated in hospital (Fixed) | 3% | Vongphoumy 2015^9^ | 3% | Vongphoumy 2015^9^ |
| Probability of digit amputation due to snakebite envenoming (Beta) | 5%  (1-16%) | Vongphoumy 2016^10^ | 5%  (1-16%) | Vongphoumy 2016^10^ |
| Probability of limb amputation due to snakebite envenoming (Beta) | 2% (0.1-12%) | Vongphoumy 2016^10^ | 4%  (0.1-12%) | Expert opinion,  Vongphoumy 2016^10^ |
| Disability weight for victims not indicated for antivenom treatment (Beta) | 0.006  (0.002-0.012) | Salomon 2013^28^ | 0.006  (0.002-0.012) | Salomon 2013^28^ |
| Disability weight for victims indicated for antivenom treatment (Beta) | 0.163  (0.109-0.227) | Salomon 2013^28^ | 0.163  (0.109-0.227) | Salomon 2013^28^ |
| Disability weight for digit amputation (Beta) | 0.005  (0.002-0.010) | Salomon 2013^28^ | 0.005  (0.002-0.010) | Salomon 2013^28^ |
| Disability weight for limb amputation (Beta) | 0.039  (0.024-0.059) | Salomon 2013^28^ | 0.039  (0.024-0.059) | Salomon 2013^28^ |
| Length of stay for victims not indicated for antivenom treatment, day (Fixed) | 1 | Expert opinion | 1 | Expert opinion |
| Length of stay for victims indicated for antivenom treatment, day (Gamma) | 5  (4-6) | Expert opinion,  Shafie 2020^29^ | 5  (4-6) | Expert opinion,  Shafie 2020^29^ |
| Number of relatives or family members who companied snakebite victims (Fixed) | 4.9 | Lao Statistic Bureau^27^ | 5.5 | Lao Statistic Bureau^27^ |
| Annual income per household | 2,979.08 | Lao Statistic Bureau^24^ | 1,878.70 | Lao Statistic Bureau^24^ |
| Annual income per person, USD (Fixed) | 1,614.65 | Lao Statistic Bureau^18^ | 1,500.86 | Lao Statistic Bureau^18^ |
| Out-of-pocket health, % of total expenditure on health (Fixed) | 41.8% | Expert opinion, World Bank 2019^17^ | 41.8% | Expert opinion, World Bank 2019^17^ |
| Discount rate on costs and outcomes (Fixed) | 3% | Attema 2018^31^ | 3% | Attema 2018^31^ |
| Traditional treatment costs, USD (Fixed) | 53.57 | Blessmann 2010^11^ | 53.57 | Blessmann 2010^11^ |

**Note:** 1 United States Dollar = 14,035.23 Laotian Kip.
