# Supplementary material for: Estimating the distributional impact of improving access to snake antivenom in urban and rural Lao People’s Democratic Republic: An extended cost-effectiveness analysis
Source: PLoS Negl Trop Dis. 2026 Jun 4;20(6):e0014420. doi: 10.1371/journal.pntd.0014420 (PMC13268137; doi:10.1371/journal.pntd.0014420)
Supplement: S3 Table — (DOCX) [file pntd.0014420.s003.docx]

**S3 Table: Micro-costing of Hospitalization costs for snakebite victims with systemic envenoming**

| **Item** | **Urban areas** | | | **Rural areas** | |
| --- | --- | --- | --- | --- | --- |
|  | **Quantity** | **Price (USD)** | **Cost (USD)** | **Adjustment (Services in rural are 20% more expensive based on expert opinion)** | **Cost (USD)** |
| **Hospitalization costs, TOTAL** |  |  | **212.54** | **1.2** | **255.04** |
| **Inpatient department services** (including healthcare providers-related costs, but excluding laboratory tests, tetanus toxoid, wound dressing, and antivenom treatment) | **5** | **9.97** | **49.87** |  |  |
| **Laboratory for systemic envenoming, average** |  |  |  |  |  |
| - Coagulation profile | 7 | 4.99 | 34.91 |  |  |
| - Complete blood count | 4 | 3.56 | 14.25 |  |  |
| - Urine analysis | 6 | 1.78 | 10.69 |  |  |
| - Electrolyte | 6 | 4.99 | 29.92 |  |  |
| - Blood urea nitrogen | 6 | 3.56 | 21.37 |  |  |
| - Creatinine | 6 | 1.42 | 8.55 |  |  |
| - Creatine kinase | 6 | 3.56 | 21.37 |  |  |
| **Tetanus toxoid** |  |  |  |  |  |
| - Tetanus toxoid | 1 | 3.49 | 3.49 |  |  |
| - Needle | 1 | 0.07 | 0.07 |  |  |
| - Syringe | 1 | 0.21 | 0.21 |  |  |
| **Wound dressing** | **5** | **3.56** | **17.81** |  |  |

**Source:** Expert opinion and local price. **Note:** 1 United States Dollar = 14,035.23 Laotian Kip (LAK).
