# Supplementary material for: Estimating the distributional impact of improving access to snake antivenom in urban and rural Lao People’s Democratic Republic: An extended cost-effectiveness analysis
Source: PLoS Negl Trop Dis. 2026 Jun 4;20(6):e0014420. doi: 10.1371/journal.pntd.0014420 (PMC13268137; doi:10.1371/journal.pntd.0014420)
Supplement: S4 Table — (DOCX) [file pntd.0014420.s004.docx]

**S4 Table: Micro-costing of Hospitalization costs for victims without snakebite envenoming**

| **Item** | **Urban areas** | | | **Rural areas** | |
| --- | --- | --- | --- | --- | --- |
|  | **Quantity** | **Price (USD)** | **Cost (USD)** | **Adjustment (Services in rural are 20% more expensive based on expert opinion)** | **Cost (USD)** |
| **Hospitalization costs, TOTAL** |  |  | **49.73** | **1.2** | **59.68** |
| **Inpatient department services** (including healthcare providers-related costs, but excluding laboratory tests, tetanus toxoid, wound dressing, and antivenom treatment) | **1** | **9.97** | **9.97** |  |  |
| **Laboratory for systemic envenoming, average** |  |  |  |  |  |
| - Coagulation profile | 2 | 4.99 | 9.97 |  |  |
| - Complete blood count | 2 | 3.56 | 7.12 |  |  |
| - Urine analysis | 1 | 1.78 | 1.78 |  |  |
| - Electrolyte | 1 | 4.99 | 4.99 |  |  |
| - Blood urea nitrogen | 1 | 3.56 | 3.56 |  |  |
| - Creatinine | 1 | 1.42 | 1.42 |  |  |
| - Creatine kinase | 1 | 3.56 | 3.56 |  |  |
| **Tetanus toxoid** |  |  |  |  |  |
| - Tetanus toxoid | 1 | 3.49 | 3.49 |  |  |
| - Needle | 1 | 0.07 | 0.07 |  |  |
| - Syringe | 1 | 0.21 | 0.21 |  |  |
| **Wound dressing** | 1 | **3.56** | **3.56** |  |  |

**Source:** Expert opinion and local price. **Note:** 1 United States Dollar = 14,035.23 Laotian Kip (LAK).
