# Supplementary material for: Estimating the distributional impact of improving access to snake antivenom in urban and rural Lao People’s Democratic Republic: An extended cost-effectiveness analysis
Source: PLoS Negl Trop Dis. 2026 Jun 4;20(6):e0014420. doi: 10.1371/journal.pntd.0014420 (PMC13268137; doi:10.1371/journal.pntd.0014420)
Supplement: S5 Table — (DOCX) [file pntd.0014420.s005.docx]

**S5 Table: Micro-costing of Antivenom treatment costs**

| **Item** | **Urban areas** | | | **Rural areas** | |
| --- | --- | --- | --- | --- | --- |
|  | **Quantity** | **Price (USD)** | **Cost (USD)** | **Adjustment (Services in rural are 20% more expensive based on expert opinion)** | **Cost (USD)** |
| **Antivenom treatment costs, total** |  |  | **431.70** | **1.2** | **518.04** |
| **Antivenom, average** | **5** | **85.50** | **427.50** |  |  |
| **Antivenom administration** |  |  |  |  |  |
| - Needle | 1 | 0.07 | 0.07 |  |  |
| - Syringe | 1 | 0.21 | 0.21 |  |  |
| - 0.9% NaCl 100 mL | 2 | 1.42 | 2.85 |  |  |
| - IV set | 1 | 1.07 | 1.07 |  |  |

**Source:** Expert opinion and local price. **Note:** 1 United States Dollar = 14,035.23 Laotian Kip (LAK).
