# Supplementary material for: Estimating the distributional impact of improving access to snake antivenom in urban and rural Lao People’s Democratic Republic: An extended cost-effectiveness analysis
Source: PLoS Negl Trop Dis. 2026 Jun 4;20(6):e0014420. doi: 10.1371/journal.pntd.0014420 (PMC13268137; doi:10.1371/journal.pntd.0014420)
Supplement: S6 Table — (DOCX) [file pntd.0014420.s006.docx]

**S6 Table: Micro-costing of Adverse reaction management costs**

| **Item** | **Urban areas** | | | **Rural areas** | |
| --- | --- | --- | --- | --- | --- |
|  | **Quantity** | **Price (USD)** | **Cost (USD)** | **Adjustment (Services in rural are 20% more expensive based on expert opinion)** | **Cost (USD)** |
| **Adverse reaction management costs*, total** |  |  | **1.92** | **1.2** | **2.31** |
| Adrenaline | 1 | 0.57 | 0.57 |  |  |
| Dexamethasone | 1 | 0.78 | 0.78 |  |  |
| - Needle | 2 | 0.07 | 0.14 |  |  |
| - Syringe | 2 | 0.21 | 0.43 |  |  |

*Adverse reaction management costs were applied to only those who experienced a hypersensitivity reaction after receiving antivenom treatment. **Source:** Expert opinion and local price. **Note:** 1 United States Dollar = 14,035.23 Laotian Kip (LAK).
