# Supplementary material for: Estimating the distributional impact of improving access to snake antivenom in urban and rural Lao People’s Democratic Republic: An extended cost-effectiveness analysis
Source: PLoS Negl Trop Dis. 2026 Jun 4;20(6):e0014420. doi: 10.1371/journal.pntd.0014420 (PMC13268137; doi:10.1371/journal.pntd.0014420)
Supplement: S7 Table — (DOCX) [file pntd.0014420.s007.docx]

**S7 Table: Micro-costing of Other Costs**

| **Item** | **Urban areas** | | | **Rural areas** | |
| --- | --- | --- | --- | --- | --- |
|  | **Quantity** | **Price (USD)** | **Cost (USD)** | **Adjustment (Services in rural are 20% more expensive)** | **Cost (USD)** |
| Digit amputation costs | 1 | 32.06 | 32.06 | 1.2 | 38.47 |
| Limb amputation costs | 1 | 97.97 | 97.97 | 1.2 | 117.56 |
| Transportation costs, round trip per family | 1 | 21.37 | 21.37 |  | 28.50 |
| Additional food costs, per day per person | 1 | 4.27 | 4.27 |  | 2.14 |

**Source:** Expert opinion and local price. **Note:** 1 United States Dollar = 14,035.23 Laotian Kip (LAK).
