# Supplementary material for: Estimating the distributional impact of improving access to snake antivenom in urban and rural Lao People’s Democratic Republic: An extended cost-effectiveness analysis
Source: PLoS Negl Trop Dis. 2026 Jun 4;20(6):e0014420. doi: 10.1371/journal.pntd.0014420 (PMC13268137; doi:10.1371/journal.pntd.0014420)
Supplement: S8 Table — (DOCX) [file pntd.0014420.s008.docx]

**S8 Table: Two-way sensitivity analyses of the impact of access to antivenom and treatment-seeking behavior on out-of-pocket expenditures for snakebite per monthly household income in urban and rural Lao PDR**

|  | **Urban** | | | | | | | **Rural** | | | | |
| --- | --- | --- | --- | --- | --- | --- | --- | --- | --- | --- | --- | --- |
| **%OOP/monthly household income** | **Relative increase in switching from traditional to conventional treatments** | | | | | | | | | | | |
| **Relative increase from  current to full access to antivenom** |  | **0** | **0.25** | **0.5** | **0.75** | **1** | **0** | | **0.25** | **0.5** | **0.75** | **1** |
|  | **0** | 20% | 26% | 31% | 36% | 41% | 0.4% | | 17% | 32% | 47% | 63% |
|  | **0.25** | 22% | 28% | 34% | 39% | 45% | 0.4% | | 19% | 36% | 53% | 71% |
|  | **0.5** | 24% | 30% | 36% | 43% | 49% | 0.4% | | 22% | 41% | 60% | 79% |
|  | **0.75** | 26% | 33% | 39% | 46% | 52% | 0.4% | | 24% | 45% | 66% | 87% |
|  | **1** | 28% | 35% | 42% | 49% | 56% | 0.4% | | 26% | 49% | 72% | 95% |

**Note:** Differences were calculated using unrounded model outputs. Values presented are rounded for readability; therefore, arithmetic differences based on displayed values may not exactly match the reported differences.
