# Supplementary material for: Estimating the distributional impact of improving access to snake antivenom in urban and rural Lao People’s Democratic Republic: An extended cost-effectiveness analysis
Source: PLoS Negl Trop Dis. 2026 Jun 4;20(6):e0014420. doi: 10.1371/journal.pntd.0014420 (PMC13268137; doi:10.1371/journal.pntd.0014420)
Supplement: S9 Table — (DOCX) [file pntd.0014420.s009.docx]

**S9 Table: Two-way sensitivity analyses of the impact of transportation cost and coverage of direct medical costs on out-of-pocket expenditures for snakebite per monthly household income in urban and rural Lao PDR**

|  | **Urban** | | | | | | | **Rural** | | | | |
| --- | --- | --- | --- | --- | --- | --- | --- | --- | --- | --- | --- | --- |
| **%OOP/monthly household income** | **Coverage of direct medical costs** | | | | | | | | | | | |
| **Transportation costs** |  | **1** | **0.75** | **0.5** | **0.25** | **1** | **1** | | **0.75** | **0.5** | **0.25** | **0** |
|  | **1** | 27% | 44% | 62% | 80% | 97% | 35% | | 71% | 106% | 142% | 178% |
|  | **0.75** | 25% | 42% | 60% | 77% | 95% | 30% | | 66% | 102% | 138% | 173% |
|  | **0.5** | 23% | 40% | 58% | 75% | 93% | 26% | | 61% | 97% | 133% | 169% |
|  | **0.25** | 20% | 38% | 56% | 73% | 91% | 21% | | 57% | 93% | 128% | 164% |
|  | **0** | 18% | 36% | 53% | 71% | 89% | 17% | | 52% | 88% | 124% | 160% |

**Note:** Differences were calculated using unrounded model outputs. Values presented are rounded for readability; therefore, arithmetic differences based on displayed values may not exactly match the reported differences.
