# Supplementary material for: Estimating the distributional impact of improving access to snake antivenom in urban and rural Lao People’s Democratic Republic: An extended cost-effectiveness analysis
Source: PLoS Negl Trop Dis. 2026 Jun 4;20(6):e0014420. doi: 10.1371/journal.pntd.0014420 (PMC13268137; doi:10.1371/journal.pntd.0014420)
Supplement: Attachment — Submitted filename: R1_Author response letter_ECEA Snakebite Lao PDR.docx [file pntd.0014420.s011.docx]

**AUTHOR RESPONSE LETTER**

**Journal: PLOS Neglected Tropical Diseases**

**Manuscript ID: PNTD-D-25-01152**

**Title: Estimating the Distributional Impact of Improving Access to Snake Antivenom in Urban and Rural Lao People's Democratic Republic: An Extended Cost-Effectiveness Analysis**

Dear Editor,

On behalf of all the authors, I would like to resubmit our revised manuscript entitled: *“Estimating the Distributional Impact of Improving Access to Snake Antivenom in Urban and Rural Lao People's Democratic Republic: An Extended Cost-Effectiveness Analysis” (PNTD-D-25-01152).* We sincerely appreciate the editors' and reviewers' comments. We have revised our manuscript and addressed each point raised by the reviewers and editors. Additionally, we adjusted our manuscript to meet the journal's requirements. We value the reviewers' insightful feedback and have made every effort to incorporate their suggestions into this revised version.

Thank you for considering this paper for publication.

Sincerely yours,

Nathorn Chaiyakunapruk, Pharm.D., Ph.D.

Department of Pharmacotherapy, University of Utah College of Pharmacy

Email: [Nathorn.Chaiyakunapruk@utah.edu](mailto:Nathorn.Chaiyakunapruk@utah.edu)

**Response to Additional Editor Comments**

In this study, authors intended to estimate the Distributional Impact of Improving Access to Snake Antivenom in Urban and Rural Lao People's Democratic Republic: An Extended Cost-Effectiveness Analysis. This manuscript addresses the public health and economic burden of snakebite envenoming in a resource-limited setting, a highly relevant and critical issue within the scope of PNTD. However, the manuscript, in its current form, requires substantial revisions to meet the expected methodological rigor and clarity.

**Response**

We would like to thank you for your valuable comments and suggestions. We have revised our manuscript and responded point-by-point to each issue you raised.

**Comment 1**

Major Comments:

Robustness of Input Parameters (Methods, lines 155–158; Limitations, lines 349–352):

The Input Parameters used for the 2023 estimates of snakebite incidence in urban and rural Lao PDR (Table S2) rely heavily on assumptions, expert opinion, and outdated data—some dating back to 2010 and 2013. This represents a major methodological vulnerability, particularly given the rapidly evolving demographic and health system context of Lao PDR. The authors should substantially expand their description of the quality, representativeness, and vintage of the “publicly available data” used to populate the model.

Specifically, the manuscript should identify:

• The exact government websites or databases consulted.

• The geographical coverage, sampling frame, and representativeness of the “published literature.”

• The number and profile of “local stakeholders” involved, including their areas of expertise.

• The procedures used to elicit expert opinion (structured interviews, Delphi rounds, group discussions, etc.) and the method of aggregation (consensus, mean, median, or other).

If expert elicitation contributed meaningfully to parameter estimation, the authors should provide concrete examples and a transparent, reproducible description of the elicitation methodology.

**Response 1**

We accepted the data constraints we had, as the data relied heavily on assumptions, expert opinion, and outdated studies. However, we tried our best to capture all available information and studies on snakebite in Lao PDR, including 4 observational studies published in 2010, 2015, 2016, and 2023.^1-4^ Apart from these four, there are case reports with limited information that cannot be used in our study.^5-7^ Therefore, we revised our manuscript to elaborate on the source of input parameters and how we obtained them. Please see the changes below.

**Reference**

1.	Blessmann J, Khonesavanh C, Outhaithit P, Manichanh S, Somphanthabansouk K, Siboualipha P. Venomous snake bites in Lao PDR: a retrospective study of 21 snakebite victims in a provincial hospital. Southeast Asian journal of tropical medicine and public health. 2010;41(1):195.

2.	Vongphoumy I, Phongmany P, Sydala S, Prasith N, Reintjes R, Blessmann J. Snakebites in two rural districts in Lao PDR: community-based surveys disclose high incidence of an invisible public health problem. PLoS neglected tropical diseases. 2015;9(6):e0003887.

3.	Vongphoumy I, Chanthilat P, Vilayvong P, Blessmann J. Prospective, consecutive case series of 158 snakebite patients treated at Savannakhet provincial hospital, Lao People's Democratic Republic with high incidence of anaphylactic shock to horse derived F (ab') 2 antivenom. Toxicon. 2016;117:13-21.

4.	Blessmann J, Hanlodsomphou S, Santisouk B, Krumkamp R, Kreuels B, Ismail AK, et al. Experience of using expired lyophilized snake antivenom during a medical emergency situation in Lao People's Democratic Republic––A possible untapped resource to tackle antivenom shortage in Southeast Asia. Tropical Medicine & International Health. 2023;28(1):64-70.

5.	Huang F, Zhao S, Tong F, Liang Y, Le Grange JM, Kuang W, et al. Unexpected death in a young man associated with a unilateral swollen leg: Pathological and toxicological findings in a fatal snakebite from Deinagkistrodon acutus (Chinese moccasin). J Forensic Sci. 2021;66(2):786-92.

6.	Ralph R, Garg D, Balachandran A, Ganesh SR, Lamb T. A case report of Ovophis monitcola (Mountain pit-viper) envenoming in northeastern India resulting in prolonged coagulopathy. Toxicon. 2023;229:107147.

7.	Blessmann J, Santisouk B, Rahden P, Kreuels B, Hanlodsomphou S. Severe local and systemic haematotoxic envenoming caused by a Malayan pit viper (Calloselasma rhodostoma) in a pregnant woman in Lao People's Democratic Republic. Toxicon. 2025;254:108227.

| **Original version** | **Revised version** |
| --- | --- |
| Methods, Input parameters, Lines 154-158  Input parameters specific to urban and rural settings were sourced from the prior CEA model^20^ and publicly available data, including published literature and government websites.^9,10,17,18,24-31^ In cases where input parameters were unavailable, we engaged local stakeholders to obtain them. Input parameters are listed in Table S2 in the supplementary material. | Methods, Input parameters, Lines 163-179  Input parameters specific to urban and rural settings were sourced from the prior CEA model^20^ and publicly available data, including published literature and government websites.^9,10,17,18,23,24,26-31^ Specifically, we searched PubMed for published articles on snakebite in the Lao PDR, complemented by Google and Google Scholar searches. Websites of the Ministry of Health of the Lao PDR and the Lao Statistics Bureau were also searched. The search period ended on 30 April 2023. All documents and websites with information on snakebites in the Lao PDR were included, and relevant details were extracted to serve as input parameters for the model. Candidate input parameters were initially identified from published literature, government databases, and official websites by three researchers (C.P., J.C., and N.C.). This process was followed by a virtual group discussion with three researchers (M.M., K.S., and J.B.) who have extensive experience in the clinical management and epidemiology of snakebite envenoming in Lao PDR, to evaluate their relevance and suitability in the Lao PDR context. When data were unavailable, expert opinion was used to estimate plausible parameter values. We validated assumptions through multiple sources and adopted conservative estimates whenever possible. Input parameters are listed in Table S2 in the supplementary material. |
| Discussion, Limitations, Lines 349-352  This ECEA should be interpreted with the following limitations. Firstly, input parameters were derived from a handful of published articles, and when data were unavailable, we relied on expert opinion. To address the inherent uncertainty in these input parameters, we validated our assumptions using multiple sources and adopted conservative estimates where possible. | Discussion, Limitations, Lines 384-388  This ECEA should be interpreted with the following limitations. Firstly, input parameters were derived from a handful of published observational studies of snakebites in the Lao PDR.^9-12^ In addition, when data were unavailable, we relied on expert opinion. To address the inherent uncertainty in these input parameters, we validated our assumptions using multiple sources and adopted conservative estimates where possible. |

**Comment 2**

Table S3 – Micro costing of Hospitalization Costs:

The costing of hospitalization for systemic envenoming appears incomplete. Important complications—such as secondary infections, surgical interventions, and renal replacement therapy—are not included, despite occurring in more than 25% of cases in many settings. Their omission likely leads to a substantial underestimation of true hospitalization costs.

**Response 2**

We agreed that these complications should have been incorporated into our model. However, due to the lack of empirical evidence in Lao PDR, we decided to adopt conservative estimates where possible by including only amputation. We acknowledged that omitting these complications and disabilities could underestimate the burden of snakebites in Lao PDR. Therefore, we revised our manuscript to elaborate on this point. Please see the changes below.

| **Original version** | **Revised version** |
| --- | --- |
| Discussion, Limitations, Lines 353-357  Secondly, while snakebite envenoming can result in multiple physical and mental disabilities,^35,36^ our model included only amputations, as they are the only disability of snakebite, for which we had sufficient evidence to incorporate into the model.^10^ This constraint underscores the need for further research to capture the broader spectrum of snakebite-related disabilities in Laos. | Discussion, Limitations, Lines 388-395  Secondly, while snakebite envenoming can result in multiple physical and mental complications and disabilities, such as secondary infections, surgical complications, acute kidney injuries, post-traumatic stress disorder, or anxiety,^35,36^ our model included only amputations, as they are the only disability of snakebite, for which we had sufficient evidence to incorporate into the model.^10^ Omitting these complications and disabilities could underestimate the burden of snakebites and the impact of improving access to antivenom in Lao PDR, and this constraint underscores the need for further research to capture the broader spectrum of snakebite-related disabilities in Lao PDR. |

**Comment 3**

Additionally, the rationale for including six urine analyses is unclear. The authors should clarify the clinical management protocol for snakebite envenoming in Lao PDR, including how diagnostic and monitoring practices vary by severity grade.

**Response 3**

Through group discussion, three local clinicians (M.M., K.S., and J.B.) estimated the average resource utilization for managing snakebite patients in the context of Lao PDR, which was further used for the micro-costing approach. This was done due to the lack of empirical data on the economic burden of snakebites in Lao PDR. Therefore, we revised our manuscript to elaborate on this point. Please see the changes below.

| **Original version** | **Revised version** |
| --- | --- |
| Methods, Costs, Lines 170-171  A micro-costing approach was employed to estimate the costs associated with snakebite (Tables S3-7 in the supplementary material). | Methods, Costs, Lines 192-195  A micro-costing approach was employed to estimate the costs associated with snakebite. Through group discussion, three local clinicians (M.M., K.S., and J.B.) estimated the average resource utilization for managing snakebite patients in the context of Lao PDR (Tables S3-7 in the supplementary material). |

**Comment 4**

Table S7 – Micro costing of Other Costs:

It is unclear whether human resource costs (nursing, medical, anesthesiology, pharmacist, etc.) were incorporated. These typically represent a major component of hospital expenditures and should be explicitly addressed.

**Response 4**

These human resource costs were already bundled under the costs for inpatient department services. Therefore, we have noted in Tables S3 and S4 in the Supplementary material that “Inpatient department services (including healthcare providers-related costs, but excluding laboratory tests, tetanus toxoid, wound dressing, and antivenom treatment).”

**Comment 5**

The authors should also clarify whether inter facility transfers or medical evacuations—from rural to urban centers for higher level care—were included in the costing framework.

**Response 5**

Transportation costs were assumed to include only cars and motorcycles. Inter-facility transportation, medical evacuation, and ambulance cars were not captured in the costing framework. Therefore, we revised our manuscript to elaborate on this point. Please see the changes below.

| **Original version** | **Revised version** |
| --- | --- |
| Methods, Costs, Lines 173-175  Direct non-medical costs included transportation and additional food expenses for patients and their caregivers. | Methods, Costs, Lines 197-200  Direct non-medical costs included transportation and additional food expenses for patients and their caregivers. Transportation costs were assumed to include only cars and motorcycles. Inter-facility transportation, medical evacuation, and ambulance cars were not captured in the costing framework. |

**Comment 6**

Table S2 – Input Parameters:

Several references cited in Table S2 do not correspond to the reference list or numbering in the main manuscript. This discrepancy must be corrected to ensure traceability and reproducibility. **Response 6**

We have corrected the reference numbering of the Supplementary material to match the reference numbering in the main manuscript.

**Comment 7**

Table S6 – Antivenom Treatment Costs:

The manuscript should specify whether the costs of managing adverse reactions were applied universally to all envenomed patients receiving antivenom, or only to the expected proportion experiencing reactions.

**Response 7**

Costs of managing adverse reactions were applied to envenomed patients receiving antivenom who experience a hypersensitivity reaction (23%), as shown in the input parameter Table S2.

We have noted this point as a footnote of Table S6 in the Supplementary material, stating that

“Adverse reaction management costs were applied to only those who experienced a hypersensitivity reaction after receiving antivenom treatment.”

**Comment 8**

Definition and Implementation of “Full Access”:

The concept of “full access” requires clearer operationalization. Beyond the provision of antivenom, does it imply the absence of geographic barriers, timely access (e.g., within 6 hours), or fully functional supply chains? Even under idealized conditions, real world constraints would likely prevent perfect coverage. The authors should explain how “100% conventional treatment” and “100% antivenom treatment” were implemented in the model, given current treatment rates of only 6.6% (urban) and 3.0% (rural). This discrepancy warrants explicit modeling or, at minimum, a dedicated sensitivity analysis.

**Response 8**

“Full access” is a hypothetical scenario in which it is assumed that, ideally, 100% of snakebite victims seek conventional treatment and that 100% of snakebite victims with envenoming receive antivenom. We agreed that this is an idealized scenario, but our findings would indicate what could occur if access to antivenom were improved in Lao PDR. Thus, we have discussed ways forward to achieve this in the Policy Implications.

Access rate was determined by the number of available antivenoms and the average number of antivenoms used per case (20 and 90 available antivenom treatments in rural and urban Lao PDR, respectively). This resulted in 6.6% (urban) and 3.0% (rural) of snakebite envenoming patients receiving antivenom treatments in the current access scenario. We also explored the impact of seeking care at the conventional healthcare facilities and improving access to antivenom in the two-way sensitivity analyses (Table S8 in the Supplementary material) Therefore, we revised our manuscript to elaborate on this point. Please see the changes below.

| **Original version** | **Revised version** |
| --- | --- |
| Methods, Overall approach, Lines 136-139  We conducted an ECEA to evaluate the distributional impact of improving access to antivenom in urban and rural areas of Laos. This evaluation compared the current scenario (limited access), where only a small proportion of victims received antivenom treatment, with an alternative “full access” scenario, where all victims were assumed to receive antivenom. | Methods, Overall approach, Lines 138-146  We conducted an ECEA to evaluate the distributional impact of improving access to antivenom in urban and rural areas of Lao PDR. Urban and rural areas were defined according to administrative classifications used in national statistics, with urban areas corresponding to provincial capitals and urban districts, and rural areas encompassing populations outside these administrative boundaries.^18,23,24^ This evaluation compared the “current access” scenario (limited access), where only a small proportion of victims received antivenom treatment in conventional healthcare facilities, with an alternative hypothetical “full access” scenario, where all victims were assumed to ideally seek conventional treatment, and all snakebite victims with systemic envenoming meeting clinical indications for antivenom treatment receive antivenom. |
| Methods, Model, Lines 148-150  Snakebite victims were modeled as seeking care from conventional healthcare facilities (public or private), traditional healers, or receiving no treatment. Antivenom treatment was provided based on the level of access at the point of care. | Methods, Model, Lines 155-159  Snakebite victims were modeled as seeking care from conventional healthcare facilities (public or private), traditional healers, or receiving no treatment. Antivenom treatment was provided based on the level of access at the conventional healthcare facilities, determined by the number of available antivenoms and the average number of antivenoms used per case (20 and 90 available antivenom treatments in rural and urban Lao PDR, respectively). |

**Comment 9**

Model Limitation – Disability Spectrum:

The statement that “our model included only amputations” substantially understates the limitation. Snakebite envenoming is associated with a broad spectrum of long term sequelae—including chronic pain, neurological deficits, psychological trauma, and persistent functional impairment—that may contribute significantly to DALYs. The authors should discuss the likely direction and magnitude of bias introduced by excluding these outcomes. A sensitivity analysis incorporating additional, more prevalent but less severe disabilities would meaningfully strengthen the robustness of the DALY estimates and the interpretation of inequities.

**Response 9**

We agreed that these complications should have been incorporated into our model. However, due to the lack of empirical evidence in Lao PDR, we decided to adopt conservative estimates where possible by including only amputation. We acknowledged that omitting these complications and disabilities could underestimate the burden of snakebites in the Lao PDR. Therefore, we revised our manuscript to elaborate on this point. Please see the changes below.

| **Original version** | **Revised version** |
| --- | --- |
| Discussion, Limitations, Line 353-357  Secondly, while snakebite envenoming can result in multiple physical and mental disabilities,^35,36^ our model included only amputations, as they are the only disability of snakebite, for which we had sufficient evidence to incorporate into the model.^10^ This constraint underscores the need for further research to capture the broader spectrum of snakebite-related disabilities in Laos. | Discussion, Limitations, Lines 388-395  Secondly, while snakebite envenoming can result in multiple physical and mental complications and disabilities, such as secondary infections, surgical complications, acute kidney injuries, post-traumatic stress disorder, or anxiety,^35,36^ our model included only amputations, as they are the only disability of snakebite, for which we had sufficient evidence to incorporate into the model.^10^ Omitting these complications and disabilities could underestimate the burden of snakebites and the impact of improving access to antivenom in Lao PDR. This constraint underscores the need for further research to capture the broader spectrum of snakebite-related disabilities in Lao PDR. |

**Comment 10**

Minor Comments

Use consistent terminology throughout. After first defining “Lao People’s Democratic Republic (PDR),” the manuscript should consistently use “Lao PDR.”

**Response 10**

The term “Laos” has been replaced with “Lao PDR” to ensure consistent use of the word.

**Comment 11**

Line 357: “Thirdlly” should be corrected to “Thirdly.”

**Response 11**

This typo has been revised.

**Comment 12**

This manuscript has the potential to make a meaningful contribution to the field. A comprehensive revision addressing the issues outlined above is strongly recommended.

**Response 12**

We would like to thank you for your valuable comments and suggestions. We have revised our manuscript and responded point-by-point to each issue you raised.

**Response to Reviewer Comments**

**Comment 13**

Methods

Reviewer #1: Method

- Line 157-158 – “In cases where input parameters were unavailable, we engaged local stakeholders to obtain them.” Would estimate be a better word than obtain here?

**Response 13**

In response to editors’ and reviewers’ comments on input parameters, this section has been revised as shown below.

| **Original version** | **Revised version** |
| --- | --- |
| Methods, Input parameters, Lines 154-158  Input parameters specific to urban and rural settings were sourced from the prior CEA model^20^ and publicly available data, including published literature and government websites.^9,10,17,18,24-31^ In cases where input parameters were unavailable, we engaged local stakeholders to obtain them. Input parameters are listed in Table S2 in the supplementary material. | Methods, Input parameters, Lines 163-179  Input parameters specific to urban and rural settings were sourced from the prior CEA model^20^ and publicly available data, including published literature and government websites.^9,10,17,18,23,24,26-31^ Specifically, we searched PubMed for published articles on snakebite in the Lao PDR, complemented by Google and Google Scholar searches. Websites of the Ministry of Health of the Lao PDR and the Lao Statistics Bureau were also searched. The search period ended on 30 April 2023. All documents and websites with information on snakebites in the Lao PDR were included, and relevant details were extracted to serve as input parameters for the model. Candidate input parameters were initially identified from published literature, government databases, and official websites by three researchers (C.P., J.C., and N.C.). This process was followed by a virtual group discussion with three researchers (M.M., K.S., and J.B.) who have extensive experience in the clinical management and epidemiology of snakebite envenoming in Lao PDR, to evaluate their relevance and suitability in the Lao PDR context. When data were unavailable, expert opinion was used to estimate plausible parameter values. We validated assumptions through multiple sources and adopted conservative estimates whenever possible. Input parameters are listed in Table S2 in the supplementary material. |

**Comment 14**

Methods

Reviewer #2: No major new analyses or experiments are required for acceptability.

**Response 14**

We would like to thank you for your valuable comments and suggestions.

**Comment 15**

Methods

Reviewer #4: The manuscript discusses cost-effectiveness and AV distribution which potentially has important information for policy makers.

I nevertheless have difficulties following the manuscript as it is unclear in the results which data is used from the team’s earlier study on cost-effectiveness treating all snakebite envenoming cases and which data has calculations or data collected for this manuscript only.

**Response 15**

This extended cost-effectiveness analysis (ECEA) adjusted the model from our earlier study, which considered all victims without differentiating between rural and urban areas. As a result, the previous model, along with its input parameters and assumptions, was modified to estimate the new findings presented in this manuscript, demonstrating the impact of improved access between rural and urban areas on the burden of snakebite for society, the health system, and households. We have elaborated on this point in the first paragraph of the Discussion. Please see the changes below.

| **Original version** | **Revised version** |
| --- | --- |
| Discussion, Lines 302-303  Our research highlighted significant health inequities between urban and rural communities in Laos concerning access to antivenom. | Discussion, Lines 330-336  Our research highlighted significant health inequities between urban and rural communities in Lao PDR concerning access to antivenom. This ECEA adjusted the model from the previous study, which considered all victims without differentiating between rural and urban areas. As a result, the previous model, along with its input parameters and assumptions, was modified to estimate the new findings presented in this ECEA, demonstrating the impact of improved access between rural and urban areas on the burden of snakebite for society, the health system, and households in Lao PDR. |

**Comment 16**

Reviewer #4: With the mortality and morbidity used as number per 100,000 inhabitants, why do the authors think the difference between rural and urban areas is based on AV access? I expect this difference to be based on an increased human/snake interaction in rural areas which is related to occupation/accommodation etc and in numbers per 100,000 inhabitants will therefore never change unless you invest in prevention. More useful approach would be to take the morbidity/mortality per snakebite victim when thinking about inequality. The mortality reduction in rural areas is an overestimate (from 2.79 to 1.27 per 100,000 population) of the impact of AV.

**Response 16**

We agree that differences in snakebite incidence between rural and urban populations are largely driven by differences in exposure related to occupation, living conditions, and human–snake interactions, and that antivenom access alone does not reduce the incidence of snakebite at the population level.

In our analysis, the observed differences in mortality and morbidity per 100,000 population between rural and urban areas are not attributed solely to differences in antivenom access. Rather, they reflect the combined effects of multiple factors explicitly incorporated into the model, including differences in snakebite incidence, treatment-seeking behavior, and antivenom availability at the healthcare facilities. Antivenom access affects outcomes conditional on snakebite occurrence, primarily by reducing disease severity and case fatality, while incidence itself remains unchanged in the model.

With respect to inequality metrics, mortality and morbidity per snakebite victim were already considered through the reporting of case fatality rates (CFRs), which capture differences in outcomes conditional on being bitten. Population-based metrics (per 100,000 population) were used in parallel, as these are central to the extended cost-effectiveness analysis framework and allow assessment of distributional health impacts at the population level.

We agree with the reviewer that prevention strategies are essential for reducing the incidence of snakebite, particularly in rural settings. We have therefore strengthened the Discussion to explicitly acknowledge the role of prevention and environmental and occupational risk reduction as complementary strategies to improvements in access to antivenom and healthcare services. We added the following sentences to the Discussion, Policy Implications, Lines 456-461: “In parallel, prevention strategies remain critical for reducing the incidence of snakebite, particularly in rural and agricultural settings. Environmental and occupational risk-reduction measures, such as community education on snake avoidance, the use of protective equipment during agricultural work, improved housing conditions, and targeted interventions in high-risk areas, should be integrated with healthcare system strengthening efforts.”

**Comment 17**

Reviewer #4: Other questions I have are:

Introduction:

Line 72/73: how essential AV if mortality is already that low and reduction of mortality is only 36% of an already low mortality rate? Does the national guideline recommend treatment with AV for all snakebite victims? Low mortality seems to justify treating only people with specific symptoms/indications.

**Response 17**

We agree that antivenom is not indicated for all snakebite victims and is recommended only for patients with systemic envenoming or specific clinical indications, in accordance with national and international treatment guidelines. We have revised the Introduction to clarify that antivenom is essential for appropriately selected patients with systemic envenoming and to avoid implying universal administration. Please see the changes below.

| **Original version** | **Revised version** |
| --- | --- |
| Introduction, Lines 68-73  The Global Burden of Disease Study 2019 identified snakebite envenoming as the most fatal neglected tropical disease (NTD), accounting for an estimated 63,400 deaths globally, corresponding to an age-standardized mortality rate (ASMR) of 0.8 per 100,000 population.^1^ Antivenom, the only specific treatment for snakebite envenoming, has been pivotal in reducing the ASMR by 36% since 1990.^1^ | Introduction, Lines 68-74  The Global Burden of Disease Study 2019 identified snakebite envenoming as the most fatal neglected tropical disease (NTD), accounting for an estimated 63,400 deaths globally, corresponding to an age-standardized mortality rate (ASMR) of 0.8 per 100,000 population.^1^ Antivenom, the only specific treatment for snakebite envenoming, is essential for preventing severe morbidity and death among patients with systemic envenoming and clinical indications, and has been pivotal in reducing the ASMR by 36% since 1990.^1^ |

**Comment 18**

Reviewer #4: Line 84: how small?

**Response 18**

Hematotoxic snakes make up about 70% of the reported cases, while neurotoxic snakes account for approximately 30%.^1-4^ Please see the changes below.

| **Original version** | **Revised version** |
| --- | --- |
| Introduction, Lines 84-85  Epidemiological studies of snakebites in the Lao PDR suggested that viperid bites constitute the majority of reported envenomings, with neurotoxic cases representing a smaller but severe subset.^9-12^ | Introduction, Lines 84-86  Epidemiological studies of snakebites in the Lao PDR suggested that viperid bites constitute approximately 70% of reported envenomings, with neurotoxic cases representing a smaller but severe subset.^9-12^ |

**Comment 19**

Reviewer #4: Line 92 so mortality is how much? If only 4.2% of victims get AV and mortality is low, it again suggests only a subset of patients actually needs AV.

**Response 19**

We agreed with your point. We have added the reported fatality rate of snakebites (up to approximately 3%) in the Introduction. However, deaths are likely to be underestimated due to limitations in hospital-based mortality data and cultural preferences for dying at home. We also elaborate on the importance of the targeted use of antivenom in the discussion. Please see the changes below.

| **Original version** | **Revised version** |
| --- | --- |
| Introduction, Line 84-85  Epidemiological studies of snakebites in the Lao PDR suggested that viperid bites constitute the majority of reported envenomings, with neurotoxic cases representing a smaller but severe subset.^9-12^ | Introduction, Line 68-74  Epidemiological studies of snakebites in the Lao PDR suggested that viperid bites constitute approximately 70% of reported envenomings, with neurotoxic cases representing a smaller but severe subset. Although reported case fatality rates in Lao PDR are relatively low (up to approximately 3%), these estimates are likely to be underestimated due to limitations in hospital-based mortality data and cultural preferences for dying at home.^9-12^ |
| Discussion, Policy implications, Lines 381-382  Policymakers should view antivenom access as a foundational but not a standalone intervention. | Discussion, Policy implications, Lines 421-425  Policymakers should view antivenom access as a foundational but not a standalone intervention. Antivenom is crucial for treating systemic envenoming, and timely use prevents severe complications and death. Due to resource limits in low-income settings such as Lao PDR, antivenom should be targeted, not universal. Improving clinical triage, following guidelines, and early detection are key to using limited supplies efficiently and equitably. Training healthcare workers to recognize indications for antivenom, alongside improvements in referral pathways and supportive care capacity, can maximize the health gains achievable from existing antivenom resources while minimizing unnecessary use. |

**Comment 20**

Reviewer #4: 106-113: this paragraph seems repetitive.

**Response 20**

We have rewritten this paragraph as suggested. Please see the changes below.

| **Original version** | **Revised version** |
| --- | --- |
| Introduction, Lines 106-113  Snakebite envenoming is widely recognized as a “disease of poverty,” disproportionately affecting socioeconomically disadvantaged populations who face significant barriers to accessing care.^19^ In Laos, snakebites are predominantly reported in rural areas, where agricultural activities and forest proximity increase exposure to snake habitats.^9,11^ These rural populations are often socially and economically disadvantaged, rendering them more vulnerable to snakebites. Access to care is further constrained by the geographic concentration of antivenoms in urban hospitals,^2^ exacerbating health inequities between urban and rural communities in Laos. | Introduction, Lines 110-115  Snakebite envenoming is widely recognized as a “disease of poverty,” disproportionately affecting socioeconomically disadvantaged populations.^19^ In Lao PDR, the burden of snakebite is concentrated in rural areas, where agricultural activities and proximity to forests increase exposure risk. These rural populations also face greater barriers to accessing care, including the concentration of antivenom supplies in urban hospitals, which exacerbates health inequities between urban and rural communities.^2,9,11^ |

**Comment 21**

Reviewer #4: 119: pleas summarize the findings of the study in ref 20 as this study compares full AV use with current AV use and it’s not clear what was added with this study. Can you in the results make clear which findings are used from ref 20 and which data was collected and analysed anew?

**Response 21**

This extended cost-effectiveness analysis (ECEA) adjusted the model from our earlier study, which considered all victims without differentiating between rural and urban areas. As a result, the previous model, along with its input parameters and assumptions, was modified to estimate the new findings presented in this manuscript, demonstrating the impact of improved access between rural and urban areas on the burden of snakebite for society, the health system, and households. We have revised the introduction to provide a summary of findings from the previous study. We also elaborated on this point in the first paragraph of the Discussion. Please see the changes below.

| **Original version** | **Revised version** |
| --- | --- |
| Introduction, Lines 116-119  Improving access to antivenom represents a crucial strategy for mitigating the burden of snakebite envenoming. A prior cost-effectiveness analysis (CEA) highlighted the substantial clinical and economic benefits of hypothetically treating all snakebite envenoming cases in Laos with antivenoms.^20^ | Introduction, Lines 117-121  Improving access to antivenom represents a crucial strategy for mitigating the burden of snakebite envenoming. A prior cost-effectiveness analysis (CEA) highlighted the substantial clinical and economic benefits of hypothetically treating all snakebite envenoming cases in Lao PDR with antivenoms, resulting in cost savings of 4,710 United States Dollars (USD), averting 0.06 deaths and 1.5 DALYs per case, thereby constituting a dominant strategy.^20^ |
| Discussion, Lines 302-303  Our research highlighted significant health inequities between urban and rural communities in Laos concerning access to antivenom. | Discussion, Lines 330-336  Our research highlighted significant health inequities between urban and rural communities in Lao PDR concerning access to antivenom. This ECEA adjusted the model from the previous study, which considered all victims without differentiating between rural and urban areas. As a result, the previous model, along with its input parameters and assumptions, was modified to estimate the new findings presented in this ECEA, demonstrating the impact of improved access between rural and urban areas on the burden of snakebite for society, the health system, and households in Lao PDR. |

**Comment 22**

Reviewer #4: Methods:

139: you don’t want to give AV to everyone; what was the definition of need for antivenom? If not exactly known, estimate around half or so depending on earlier findings how many people developed systemic envenoming versus local signs only?

**Response 22**

We have revised the Methods to clarify that antivenom is essential for appropriately selected patients with systemic envenoming. In our model, we applied probabilities of systemic envenoming for antivenom treatment of 20% in urban areas and 35% in rural areas for those who seek conventional treatment, and 20% in rural areas for those who seek conventional treatment, as shown in Table S2 in the supplementary material. Please see the changes below.

| **Original version** | **Revised version** |
| --- | --- |
| Methods, Overall approach, Lines 136-139  We conducted an ECEA to evaluate the distributional impact of improving access to antivenom in urban and rural areas of Laos. This evaluation compared the current scenario (limited access), where only a small proportion of victims received antivenom treatment, with an alternative “full access” scenario, where all victims were assumed to receive antivenom. | Methods, Overall approach, Lines 138-146  We conducted an ECEA to evaluate the distributional impact of improving access to antivenom in urban and rural areas of Lao PDR. Urban and rural areas were defined according to administrative classifications used in national statistics, with urban areas corresponding to provincial capitals and urban districts, and rural areas encompassing populations outside these administrative boundaries.^18,23,24^ This evaluation compared the “current access” scenario (limited access), where only a small proportion of victims received antivenom treatment in conventional healthcare facilities, with an alternative hypothetical “full access” scenario, where all victims were assumed to ideally seek conventional treatment, and all snakebite victims with systemic envenoming meeting clinical indications for antivenom treatment receive antivenom. |

**Comment 23**

Reviewer #4: Methods:

150 point of care = where?

**Response 23**

Point of care is at the conventional healthcare facilities. Please see the changes below.

| **Original version** | **Revised version** |
| --- | --- |
| Methods, Model, Lines 148-150  Snakebite victims were modeled as seeking care from conventional healthcare facilities (public or private), traditional healers, or receiving no treatment. Antivenom treatment was provided based on the level of access at the point of care. | Methods, Model, Lines 155-159  Snakebite victims were modeled as seeking care from conventional healthcare facilities (public or private), traditional healers, or receiving no treatment. Antivenom treatment was provided based on the level of access at the conventional healthcare facilities, determined by the number of available antivenoms and the average number of antivenoms used per case (20 and 90 available antivenom treatments in rural and urban Lao PDR, respectively). |

**Comment 24**

Reviewer #4: How did you define urban and rural?

**Response 24**

We have provided our definitions in the Methods. Urban and rural areas were defined according to administrative classifications used in national statistics, with urban areas corresponding to provincial capitals and urban districts, and rural areas encompassing populations outside these administrative boundaries. Please see the changes below.

| **Original version** | **Revised version** |
| --- | --- |
| Methods, Overall approach, Lines 136-139  We conducted an ECEA to evaluate the distributional impact of improving access to antivenom in urban and rural areas of Laos. This evaluation compared the current scenario (limited access), where only a small proportion of victims received antivenom treatment, with an alternative “full access” scenario, where all victims were assumed to receive antivenom. | Methods, Overall approach, Lines 138-146  We conducted an ECEA to evaluate the distributional impact of improving access to antivenom in urban and rural areas of Lao PDR. Urban and rural areas were defined according to administrative classifications used in national statistics, with urban areas corresponding to provincial capitals and urban districts, and rural areas encompassing populations outside these administrative boundaries.^18,23,24^ This evaluation compared the “current access” scenario (limited access), where only a small proportion of victims received antivenom treatment in conventional healthcare facilities, with an alternative hypothetical “full access” scenario, where all victims were assumed to ideally seek conventional treatment, and all snakebite victims with systemic envenoming meeting clinical indications for antivenom treatment receive antivenom. |

**Comment 25**

Reviewer #4: Results

225 CI of this estimate? Original obtained data for this manuscript or used from other publications?

**Response 25**

The annual number of snakebite victims is a model-based estimate derived from this study's modelling approach. These estimates were calculated by applying fixed snakebite incidence rates to urban and rural population sizes (Table S2 in the supplementary material).

**Comment 26**

Reviewer #4: 229: where does the 744,077 USD come from?

**Response 26**

The estimated total societal cost is a model-based estimate derived from this study's modelling approach, using the input parameters described in the Methods section. Total societal costs were calculated by aggregating per-victim societal costs across the estimated number of snakebite cases in each setting.

**Comment 27**

Reviewer #4: 258: rural areas higher mortality is stated but CI does not demonstrate statistical significance so does not seem to justified to state there’s a significant difference

**Response 27**

The 95% credible intervals presented in the Results were derived from probabilistic sensitivity analysis (PSA) using Monte Carlo simulation and represent the uncertainty around model-based estimates, rather than confidence intervals for hypothesis testing. We further clarified this point in the Methods. Please see the changes below.

| **Original version** | **Revised version** |
| --- | --- |
| Methods, Sensitivity analyses, Lines 194-199  Several sensitivity analyses were conducted to assess uncertainties and explore the robustness of the results. Probabilistic sensitivity analysis (PSA) was performed using a Monte Carlo simulation with 1,000 iterations randomly sampled from a distribution of input parameters from distributions such as gamma (e.g., length of hospital stay) and beta (e.g., probabilities of adverse reactions, survival outcomes, and disability weights) (Table S2 in the supplementary material). The 95% credible interval (95% CrI) for health equity impacts was estimated. | Methods, Sensitivity analyses, Lines 220-226  Several sensitivity analyses were conducted to assess uncertainties and explore the robustness of the results. Probabilistic sensitivity analysis (PSA) was performed using a Monte Carlo simulation with 1,000 iterations randomly sampled from a distribution of input parameters from distributions such as gamma (e.g., length of hospital stay) and beta (e.g., probabilities of adverse reactions, survival outcomes, and disability weights) (Table S2 in the supplementary material). The 95% credible interval (95% CrI) for health equity impacts was estimated from the PSA to reflect the range of uncertainty in the model findings. |

**Comment 28**

Reviewer #1: Results

- Some of the presentation is a bit confusing, I think because of the different perspectives used, e.g. it is stated that there higher cost per patient in rural areas despite less treatment? Hospital cases in urban areas faced OOP expenditures of 102% income, 169% in rural areas. But then – “Under the current access scenario, snakebite victims in rural areas experienced worse epidemiological outcomes but lower economic burdens compared to urban areas.”

**Response 28**

Thanks for pointing out this mistake. Victims in rural areas experienced a greater economic burden despite receiving less treatment because their household incomes are substantially lower. As a result, even smaller out-of-pocket payments represented a larger share of monthly household income, as reflected in the %OOP per monthly household income measure. We have revised our manuscript that “Under the current access scenario, snakebite victims in rural areas experienced worse epidemiological outcomes **and higher** economic burdens compared to urban areas.” We also elaborated on this point in the Discussion. Please see the changes below.

| **Original version** | **Revised version** |
| --- | --- |
| Results, Lines 247-248  Under the current access scenario, snakebite victims in rural areas experienced worse epidemiological outcomes but lower economic burdens compared to urban areas. | Results, Lines 273-274  Under the current access scenario, snakebite victims in rural areas experienced worse epidemiological outcomes and higher economic burdens compared to urban areas. |
| Discussion, Lines 319-321  These costs were disproportionately higher in rural areas, with less developed healthcare infrastructure. | Discussion, Lines 352-356  These costs were disproportionately higher in rural areas, with less developed healthcare infrastructure. As household incomes in rural areas are substantially lower than urban areas, smaller OOP payments represented a larger share of monthly household income, as reflected in the %OOP per monthly household income. |

**Comment 29**

Reviewer #1: Given the uncertainty it might be a bit strong to conclude that “Rural areas had higher… household economic burden (+1% OOP expenditures per monthly household income, 95% CrI -4% to +6%), than urban areas.” Similar applies to other interpretations throughout this section, e.g. “Rural areas exhibited higher mortality rates (+1.27 deaths per 100,000 population; 95% CrI -0.95 to +7.09) and greater DALYs lost (+32.14 DALYs per 100,000 population; 95% CrI -24.23 to +193.16) compared to urban areas.”

**Response 29**

We reported our findings based on the base-case value. However, we understood the reviewer’s concern regarding the uncertainty. Therefore, we have addressed this point in the abstract and results section. Please see the changes below.

| **Original version** | **Revised version** |
| --- | --- |
| Abstract, Lines 37-44  Results: Rural areas had higher mortality (3.14 vs. 0.35, +2.79 per 100,000 population), DALYs lost (78.97 vs. 8.86, +70.11 per 100,000 population), and household OOP expenses (39% vs. 38%, +1% of household monthly income) than urban areas. Full access to antivenom reduced health inequities in mortality (from +2.79 to +1.27 per 100,000 population) and DALYs (from +70.11 to +32.14 per 100,000 population) but increased inequities in household OOP expenses (from +1% to +39% of household monthly income). | Abstract, Lines 36-44  Results: Our model indicated that rural areas had higher mortality (3.14 vs. 0.35, +2.79 per 100,000 population), DALYs lost (78.97 vs. 8.86, +70.11 per 100,000 population), and household OOP expenses (39% vs. 38%, +1% of household monthly income) than urban areas. Full access to antivenom reduced health inequities in mortality (from +2.79 to +1.27 per 100,000 population) and DALYs (from +70.11 to +32.14 per 100,000 population) but increased inequities in household OOP expenses (from +1% to +39% of household monthly income). These differences had considerable uncertainties. |
| Results, Lines 247-263  Under the current access scenario, snakebite victims in rural areas experienced worse epidemiological outcomes but lower economic burdens compared to urban areas. Rural areas had higher mortality rates (+2.79 deaths per 100,000 population; 95% CrI +0.40 to +4.29), DALYs lost (+70.11 DALYs per 100,000 population; 95% CrI +12.04 to +105.47) and household economic burden (+1% OOP expenditures per monthly household income, 95% CrI -4% to +6%), than urban areas.  Improving access to antivenom reduced health inequities in deaths and DALYs lost between urban and rural areas. However, it exacerbated health inequities in household economic burden. In the full access scenario, while rural areas still showed worse epidemiological outcomes than urban areas, the health inequities in mortality and DALYs lost were reduced. Rural areas exhibited higher mortality rates (+1.27 deaths per 100,000 population; 95% CrI -0.95 to +7.09) and greater DALYs lost (+32.14 DALYs per 100,000 population; 95% CrI -24.23 to +193.16) compared to urban areas. The household economic burden in rural areas increased significantly compared to urban areas (95% vs. 56% of OOP expenditures per monthly household income), with 39% higher OOP expenditures per monthly household income (95% CrI +23% to +52%). These findings are presented in Table 2 and Figure 1. | Results, Lines 273-291  Under the current access scenario, snakebite victims in rural areas experienced worse epidemiological outcomes and higher economic burdens compared to urban areas. Rural areas had higher mortality rates (+2.79 deaths per 100,000 population; 95% CrI +0.40 to +4.29), DALYs lost (+70.11 DALYs per 100,000 population; 95% CrI +12.04 to +105.47) and household economic burden (+1% OOP expenditures per monthly household income, 95% CrI -4% to +6%), than urban areas. These differences were associated with considerable uncertainty, as reflected in the wide credible intervals.  Improving access to antivenom reduced health inequities in deaths and DALYs lost between urban and rural areas. However, it exacerbated health inequities in the household economic burden. In the full access scenario, while rural areas still showed worse epidemiological outcomes than urban areas, the health inequities in mortality and DALYs lost were reduced. Rural areas exhibited higher mortality rates (+1.27 deaths per 100,000 population; 95% CrI -0.95 to +7.09) and greater DALYs lost (+32.14 DALYs per 100,000 population; 95% CrI -24.23 to +193.16) compared to urban areas. The household economic burden in rural areas increased significantly compared to urban areas (95% vs. 56% of OOP expenditures per monthly household income), with 39% higher OOP expenditures per monthly household income (95% CrI +23% to +52%). Wide credible intervals indicated considerable uncertainty about these differences. These findings are presented in Table 2 and Figure 1. |

**Comment 30**

Reviewer #1: “Improving access to antivenom reduced health inequities in deaths and DALYs lost between urban and rural areas. However, it exacerbated health inequities in household economic burden.” Is the latter of this based on OOP of patients or overall incidences of CHE?

**Response 30**

This is based on the %OOP per monthly houshold income as reported in the manuscript that The household economic burden in rural areas increased significantly compared to urban areas (95% vs. 56% of OOP expenditures per monthly household income), with 39% higher OOP expenditures per monthly household income (95% CrI +23% to +52%).

**Comment 31**

Reviewer #1: Why is out of pocket cost presented as a percentage per patient? While it is an important measure (presumably particularly for the patient) that should be reported, I would have thought that maybe a measure of the numbers of catastrophic health expenditure cases under each scenario would be the better measure to compare the relative impact across the two groups (or probably CHE per 100,000 population to match the other outcomes presented)? Why have the authors chosen to present the data in this way?

**Response 31**

We agree that estimating the incidence of CHE would provide an important measure of financial risk protection. However, the objective of this analysis was to compare relative economic burden between urban and rural populations within an ECEA framework rather than to model income-distribution–dependent financial risk metrics. The model was parameterized using average income estimates at the population level and did not simulate the household-level income heterogeneity required to estimate CHE incidence under threshold-based definitions. Given these data and structural constraints, we expressed OOP payments as a proportion of average monthly household income to capture differences in financial burden attributable to income disparities between settings. We acknowledge that this measure should be interpreted as a proxy indicator of affordability rather than a formal estimate of CHE, and we have clarified this limitation in the revised manuscript. Please see the changes below.

| **Original version** | **Revised version** |
| --- | --- |
| Sensitivity analyses, Lines 357-361  Thirdlly, we assumed that antivenom administration improves outcomes across all snakebite cases. While clinical outcomes vary by snake type and time to treatment, our model applies an average effectiveness across cases due to the lack of species-specific real-world data in Lao PDR. We acknowledge this may overestimate impact in delayed cases or for envenomings where antivenom is less effective. | Discussion, Limitations, Lines 395-402  Thirdly, we assumed that antivenom administration improves outcomes across all snakebite cases. While clinical outcomes vary by snake type and time to treatment, our model applies an average effectiveness across cases due to the lack of species-specific real-world data in Lao PDR. We acknowledge this may overestimate impact in delayed cases or for envenomings where antivenom is less effective. Lastly, the analysis did not incorporate household-level income distributions and therefore could not estimate the incidence of CHE. The %OOP per monthly household income should be interpreted as a proxy of financial risk protection. |

**Comment 32**

Reviewer #1: Why is the change in mortality rate for the rural population so uncertain? This casts doubt on the interpretation of these results, but if just applying the effect of greater access, I’m not sure why the credible interval would be so big? Why would it potentially be higher than the current situation?

**Response 32**

The width of the credible interval for the change in mortality primarily reflects uncertainty in key clinical effectiveness parameters, particularly the Probability of death in systemic envenoming treated with antivenom. This parameter was informed by limited published evidence and therefore specified with a relatively wide range (2%, range 1-16%) to reflect uncertainty in treatment outcomes in this setting.

**Comment 33**

Reviewer #2: No additional analyses are required to support the Results as presented.

**Response 33**

We thank the reviewers for their valuable comments, which will help further improve our manuscript. Please see our point-by-point responses above.

**Comment 34**

Reviewer #1: Some changes might be needed if changes are made above.

**Response 34**

We thank the reviewers for their valuable comments, which will help further improve our manuscript. Please see our point-by-point responses above.

**Comment 35**

Reviewer #4: related to more general questions which influence conclusions

**Response 35**

We thank the reviewers for their valuable comments, which will help further improve our manuscript. Please see our point-by-point responses above.

**Comment 36**

**Editorial and Data Presentation Modifications**

Reviewer #2: The manuscript is generally clear and well organized, but a few minor editorial and data-presentation issues should be addressed to improve clarity.

In Table 2 and Figure 1, some reported difference values do not match the arithmetic differences implied by the reported urban and rural point estimates. For example, under current access, the difference in %OOP/monthly household income (hospital only) between rural (87%) and urban (42%) populations is reported as +44%, whereas the arithmetic difference is +45%. Similarly, under full access, the reported difference in mortality rate per 100,000 population does not correspond to the difference implied by the reported urban and rural values (1.47 − 0.25 = +1.22, whereas the table and Figure 1 report +1.27). This inconsistency is also reflected in the corresponding figure 1.

The authors should verify these difference calculations and either correct the reported values or clarify in the table footnotes and figure captions that differences are calculated from unrounded model outputs rather than the rounded point estimates shown.

Apart from these points, no new analyses or data re-analysis are required.

**Response 36**

We agreed with this point. We have added this statement to all relevant tables and figures to enhance clarity of our findings: “Differences were calculated using unrounded model outputs. Values presented are rounded for readability; therefore, arithmetic differences based on displayed values may not exactly match the reported differences.”.

**Comment 37**

**Summary and General Comments**

Reviewer #1: Thanks for the opportunity to review this paper which presents an extended cost effectiveness analysis to examine the impacts of improved access to antivenom in Lao PDR. The work tackles an important issue both in relation to this specific issue as well as the broader debate of how equity should be incorporated into policy deliberations. I have a few comments for the authors to consider, primarily around the presentation and interpretation of their analysis, as decribed in the sections above

**Response 37**

We thank the reviewers for their valuable comments, which will help further improve our manuscript. Please see our point-by-point responses above.

**Comment 38**

Reviewer #2: This manuscript presents a model-based evaluation of the health and equity impacts of improving access to antivenom for snakebite envenoming, using an extended cost-effectiveness analysis framework. The topic is highly relevant to the field of neglected tropical diseases, as snakebite remains a major but under-addressed cause of preventable morbidity, mortality, and financial hardship in low-resource settings.

The strength of the study is its focus on both health outcomes and equity-related outcomes, including out-of-pocket expenditures and distributional effects between urban and rural populations. The use of an extended cost-effectiveness approach is appropriate for the policy questions being addressed and adds value beyond standard cost-effectiveness analyses. Overall, the modeling framework is appropriate, and the analyses are generally well executed.

The manuscript is clearly written and well structured, and the results are presented in a logical and accessible manner. The tables and figures effectively summarize the main findings, and the discussion places the results in a broader public health and policy context.

The only porblem is limited to relatively minor issues in the reporting of difference values in one table and the corresponding figure. These issues appear to be correctable through verification and clarification rather than additional analyses. They do not undermine the overall conclusions of the study.

The work appears to be original, and there are no concerns related to dual publication, research ethics, or publication ethics. The study relies on secondary data sources, and ethical considerations are appropriately addressed.

Overall, this is a policy-relevant study that fits well within the scope of the journal.

**Response 38**

We thank the reviewers for their valuable comments, which will help further improve our manuscript. Please see our point-by-point responses above.
