# Supplementary material for: Estimating the distributional impact of improving access to snake antivenom in urban and rural Lao People’s Democratic Republic: An extended cost-effectiveness analysis
Source: PLoS Negl Trop Dis. 2026 Jun 4;20(6):e0014420. doi: 10.1371/journal.pntd.0014420 (PMC13268137; doi:10.1371/journal.pntd.0014420)
Supplement: Attachment — Submitted filename: R2_Author response letter_ECEA Snakebite Lao PDR NC.docx [file pntd.0014420.s012.docx]

**AUTHOR RESPONSE LETTER**

**Journal: PLOS Neglected Tropical Diseases**

**Manuscript ID: PNTD-D-25-01152R1**

**Title: Estimating the Distributional Impact of Improving Access to Snake Antivenom in Urban and Rural Lao People's Democratic Republic: An Extended Cost-Effectiveness Analysis**

Dear Editor,

On behalf of all the authors, I would like to resubmit our revised manuscript entitled: *“Estimating the Distributional Impact of Improving Access to Snake Antivenom in Urban and Rural Lao People's Democratic Republic: An Extended Cost-Effectiveness Analysis” (PNTD-D-25-01152R1).* We sincerely appreciate the editors' and reviewers' comments. We have revised our manuscript and addressed each point raised by the reviewers. We value the reviewers' insightful feedback and have made every effort to incorporate their suggestions into this revised version.

Thank you for considering this paper for publication.

Sincerely yours,

Nathorn Chaiyakunapruk, Pharm.D., Ph.D.

Department of Pharmacotherapy, University of Utah College of Pharmacy

Email: [Nathorn.Chaiyakunapruk@utah.edu](mailto:Nathorn.Chaiyakunapruk@utah.edu)

**Response to Reviewer Comments**

**Comment 1**

Reviewer #1: In the response, the authors describe their approach as fitting within an 'ECEA' framework given they do not directly model catastrophic expenditure. This is not refleced in the paper but I think the manuscript could benefit from a more precise description of how the ECEA framework has been applied in this study (particularly around the OOP). While I do not think a traditional ICER is necessarily required for an ECEA, the authors may wish to clarify how this analysis relates to their prior CEA and which components of a full ECEA are, and are not, captured here.

**Response 1**

We have rewritten the methods as suggested to explain how the ECEA framework has been applied in this study and how this analysis relates to the prior CEA. Building on our conventional CEA, this study additionally examined the distribution of health and financial consequences across equity-relevant subgroups. In line with the ECEA framework, we evaluated: (1) health outcomes, including mortality and disability-adjusted life years (DALYs); (2) financial consequences through household out-of-pocket expenditures; and (3) distributional impacts across urban and rural populations in Lao PDR. As household-level income distribution data were unavailable, we did not directly estimate catastrophic health expenditure (CHE), poverty cases averted, or money-metric insurance value. Instead, the percentage of household out-of-pocket (OOP) expenditure relative to monthly household income was used as a proxy indicator of financial hardship and affordability. Annual household income was not used because snakebite envenoming is generally an acute episodic event with high upfront costs during the envenoming episode. Please see the changes below.

| **Original version** | **Revised version** |
| --- | --- |
| Methods, Overall approach, Lines 138-139  We conducted an ECEA to evaluate the distributional impact of improving access to antivenom in urban and rural areas of Lao PDR. | Methods, Overall approach, Lines 139-154  We conducted an ECEA to evaluate the distributional impact of improving access to antivenom in urban and rural areas of Lao PDR. Building on a previous conventional CEA,^1^ this study additionally examined the distribution of health and financial consequences across equity-relevant subgroups. This ECEA adhered to the methodological framework established by Verguet and colleagues^2^ and was reported following the Consolidated Health Economic Evaluation Reporting Standards (CHEERS) 2022 guidelines (Table S1 in the supplementary material).^3^ In line with the ECEA framework, we evaluated: (1) health outcomes, including mortality and disability-adjusted life years (DALYs); (2) financial consequences through household out-of-pocket expenditures; and (3) distributional impacts across urban and rural populations in Lao PDR. As household-level income distribution data were unavailable, we did not directly estimate catastrophic health expenditure (CHE), poverty cases averted, or money-metric insurance value. Instead, the percentage of household out-of-pocket (OOP) expenditure relative to monthly household income was used as a proxy indicator of financial hardship and affordability. Annual household income was not used because snakebite envenoming is generally an acute episodic event with high upfront costs during the envenoming episode.^4^ |

**Comment 2**

Reviewer #1: The interpretation of the OOP figure as a share of monthly expenditure is compared to thresholds (e.g. 10% from the Verguet paper) that tend to refer to annual figures I believe. The presentation of these results could be a bit more nuanced I think particularly as (as the authors indicate in their response), they have not modelled catastrophic expenditure but instead included this as an affordability proxy.

**Response 2**

We agreed that the interpretation of the percentage of OOP expenditure per monthly household income requires more careful presentation. In the revised manuscript, we clarified that our analysis did not directly estimate CHE, poverty cases averted, or other formal financial risk protection metrics proposed in the ECEA framework. Instead, the percentage of OOP expenditure relative to monthly household income was used as a proxy indicator of affordability-related financial hardship.

To avoid potentially misleading interpretation, we revised the manuscript to remove direct comparisons with conventional CHE thresholds (e.g., the 10% threshold), as these are typically based on annual household expenditure or income measures and are not directly comparable to our monthly affordability indicator. Please see the changes below.

| **Original version** | **Revised version** |
| --- | --- |
| Methods, Burden of snakebite, Lines 187-189  Financial risk was assessed as the percentage of household out-of-pocket (OOP) expenditure relative to monthly household income, serving as a proxy for catastrophic health expenditure (CHE).^22^ | Methods, Burden of snakebite, Lines 203-205  Financial risk was assessed as the percentage of household OOP expenditure relative to monthly household income, serving as a proxy for financial hardship and affordability. |
| Discussion, Lines 356-360  Our estimates of the full access indicate that household OOP expenditures for snakebite reached 56% and 95% of monthly household income in urban and rural areas, respectively, far exceeding the 10% threshold for CHE.^22^ Such costs pose a significant risk of driving households into, or further into, poverty, particularly in rural areas where the need for treatment is greatest. | Discussion, Lines 374-380  Our estimates of the full access indicate that household OOP expenditures for snakebite reached 56% and 95% of monthly household income in urban and rural areas, respectively. Because a single snakebite episode consumes a disproportionate share of monthly resources, these figures illustrate the severe liquidity and affordability shock that rural households would face. Such costs pose a significant risk of driving households into, or further into, poverty, particularly in rural areas where the need for treatment is greatest. |
| Discussion, Lines 364-372  Sensitivity analyses demonstrated that full coverage of snakebite treatment could mitigate financial risks and reduce health inequities between urban and rural areas. However, the coverage was insufficient to avoid the CHE as the residual household OOP expenditures incurred by transportation and food. Our scenario analyses suggest that full coverage of antivenom and snakebite treatment, along with easily accessible healthcare facilities—specifically, zero transportation costs and one family member providing care—could ultimately reduce household OOP expenditures to 6% in urban areas and 5% in rural areas, both of which are below the threshold for CHE. | Discussion, Lines 384-391  Sensitivity analyses demonstrated that full coverage of snakebite treatment could mitigate financial risks and reduce health inequities between urban and rural areas. However, the coverage was insufficient to avoid the financial hardship as the residual household OOP expenditures incurred by transportation and food. Our scenario analyses suggest that full coverage of antivenom and snakebite treatment, along with easily accessible healthcare facilities—specifically, zero transportation costs and one family member providing care—could ultimately reduce household OOP expenditures to 6% in urban areas and 5% in rural areas. |

**Comment 3**

Reviewer #2: With regards to my comment "In Table 2 and Figure 1, some reported difference values do not match the arithmetic differences implied by the reported urban and rural point estimates. For example, under current access, the difference in %OOP/monthly household income (hospital only) between rural(87%) and urban (42%) populations is reported as +44%, whereas the arithmetic difference is+45%. Similarly, under full access, the reported difference in mortality rate per 100,000population does not correspond to the difference implied by the reported urban and rural values(1.47 − 0.25 = +1.22, whereas the table and Figure 1 report +1.27). This inconsistency is also reflected in the corresponding figure 1."

The added note is helpful for explaining small discrepancies that can arise when the urban and rural values shown in the table are rounded for readability, while the reported difference is calculated from the underlying unrounded model outputs. For example, if the true values were 42.4 and 86.6, they could be displayed as 42 and 87, while the difference based on the unrounded values would be 44.2, which could reasonably be reported as 44 rather than 45. In that kind of case, the mismatch is understandable and the rounding explanation is sufficient.

However, that explanation does not seem to resolve my concern about the reported +1.27 difference for the full-access mortality rate per 100,000 population. Based on the displayed values, the arithmetic difference is 1.47 - 0.25 = 1.22. If the displayed values are rounded to two decimal places, then the underlying unrounded urban value should be approximately between 0.245 and 0.255, and the rural value should be approximately between 1.465 and 1.475. That would imply an underlying difference of roughly 1.21 to 1.23, not 1.27. So unlike the smaller discrepancies elsewhere in the table, this one appears too large to be explained by rounding alone.

For that reason, I do not think the added note fully addresses this specific issue. If the reported difference of 1.27 was obtained from a different model summary, such as a separately summarized distribution of rural-urban differences rather than direct subtraction of the displayed point estimates, that should be stated explicitly in the table and figure notes. Otherwise, this value still appears inconsistent with the reported urban and rural estimates.

**Response 3**

Thank you for carefully reviewing the consistency of the reported values. We agree that the discrepancy in the %OOP/monthly household income value was due to rounding, as noted by the reviewer. However, we sincerely apologize that the reported difference of +1.27 deaths per 100,000 population under the full-access scenario was a typographical error. The correct value is +1.22 deaths per 100,000 population, calculated as 1.47 − 0.25.

We have corrected this value in Table 2, Figure 1, the Results section, and all other relevant parts of the manuscript. We also rechecked all numerical values reported throughout the manuscript and confirmed that this was the only typographical error identified.

**Comment 4**

Reviewer #1: The authors have engaged with the reviewer/editorial suggestions which I think has led to a stronger paper.

**Response 4**

We sincerely appreciate the editors' and reviewers' comments.

**Comment 5**

Reviewer #4: Authors answered the comments from the reviewers in a very detailed way. But there answers were to add some sentences here and there but did not change the way data were analysed and/or presented. Some examples:

- comment 1: estimates rely heavily on assumptions, expert opinion and outdated data. Add procedures used to elicit expert opinion (structured interviews, Delphi rounds) and method of aggregation.

- response was to include the references used which include retrospective studies of 21 victims. And to explain the expert opinion was based on three coauthors. the paper would have been much stronger if the methods to use 'expert opinion' was a more systematic one instead of 3 coauthors sitting together. The three authors are reported to be experienced in the clinical management, but 2 of these 3 are pharmacists and 1 is a Germany based researcher. The assumptions for the manuscript are on Lao Democratic republic.

**Response 5**

We acknowledged that the approach used to elicit expert opinion in this study was not the most rigorous or formal expert elicitation method, such as structured interviews or Delphi techniques. Due to the limited availability of snakebite-related data and experts in the Lao PDR, we relied on group discussions with researchers and clinicians with direct experience in snakebite management and epidemiology in the Lao PDR context. We have further acknowledged this limitation in the revised manuscript.

We would also like to clarify the expertise of the three researchers involved in the expert discussions. M.M. and K.S. are medical doctors in the Lao PDR with direct clinical experience in managing snakebite patients. J.B., although originally from Germany, has extensive field experience in snakebite epidemiology and clinical management research in Lao PDR, including training healthcare personnel in snakebite treatment and conducting snakebite-related research in the country for many years. In addition, J.B. has been the lead or senior author of multiple key publications on snakebite envenoming in the Lao PDR that informed the evidence base for this study. Therefore, we believe these experts were highly suitable to provide context-specific input for the model assumptions and parameter estimation. Please see the changes below.

| **Original version** | **Revised version** |
| --- | --- |
| Discussion, Limitations, Lines 384-388  Firstly, input parameters were derived from a handful of published observational studies of snakebites in the Lao PDR.^9-12^ In addition, when data were unavailable, we relied on expert opinion. To address the inherent uncertainty in these input parameters, we validated our assumptions using multiple sources and adopted conservative estimates where possible. | Discussion, Limitations, Lines 403-411  Firstly, input parameters were derived from a handful of published observational studies of snakebites in the Lao PDR.^9-12^ In addition, when data were unavailable, we relied on expert opinion obtained through group discussions with researchers and clinicians experienced in the clinical management and epidemiology of snakebite envenoming in Lao PDR. However, we did not use formal expert elicitation methods, such as structured interviews or Delphi techniques, which may limit the reproducibility and methodological rigor of the parameter estimation process. To address the inherent uncertainty in these input parameters, we validated our assumptions using multiple sources and adopted conservative estimates where possible. |

**Comment 6**

comment 2: reviewer asks for including complications and long term consequences and response = only amputations included. But assumptions on the rate of complications and long term consequences are not less unreliable than the other assumptions in the work. Now looks as authors prefer to keep the main message; that more access to AV has some impact on mortality but will have more economic impact on rural populations. With complications included, that could give a different result. Risk of anaphylactic shock after AV should also be included.

**Response 6**

We thank the reviewer for raising this important point and agree that complications, long-term sequelae, and antivenom-related anaphylaxis are clinically important aspects of snakebite envenoming. We would like to clarify why these outcomes were not modeled in greater detail and why we believe their omission is unlikely to alter the main conclusions of this study.

We adopted a conservative modeling approach by including only amputations among long-term disabilities, as these were the only complications for which sufficient evidence was available in the Lao PDR context. We acknowledge that excluding additional complications and sequelae may underestimate the overall burden of snakebite and the potential benefits of improving access to antivenom. However, we do not expect this omission to reverse the direction of main findings of the study. In our model, improved access to antivenom reflects improved access to conventional healthcare facilities capable of managing systemic envenoming, including antivenom administration and supportive care. Therefore, incorporating additional complications and long-term sequelae would likely increase the estimated health burden attributable to snakebite, particularly in rural areas where delayed access to care and severe envenoming are more common. Similarly, including the costs of managing these complications would likely increase household OOP expenditures, especially among rural households that already face greater transportation barriers and limited access to healthcare. We have revised the Limitations section to clarify this point.

Regarding anaphylactic shock, adverse reactions following antivenom administration were incorporated in the model through the probability of hypersensitivity reactions among patients receiving antivenom treatment, along with their associated management costs. The base-case probability of adverse reactions (23%) was informed by published evidence by Sriapha 2022 from Thailand using the same horse-derived F(ab')₂ antivenom product used in Lao PDR. Severe anaphylactic shock was not modeled as a separate health state. We acknowledged that this simplification may underestimate some harms and costs associated with antivenom treatment. However, severe fatal reactions to antivenom are expected to be uncommon, as zero deaths due to adverse reactions were reported in the Sriapha 2022 study. We therefore do not expect separate modeling of anaphylactic shock to alter the main conclusions regarding health inequities and financial burden between urban and rural populations. Please see the changes below.

| **Original version** | **Revised version** |
| --- | --- |
| Discussion, Limitations, Lines 388-395  Secondly, while snakebite envenoming can result in multiple physical and mental complications and disabilities, such as secondary infections, surgical complications, acute kidney injuries, post-traumatic stress disorder, or anxiety,^35,36^ our model included only amputations, as they are the only disability of snakebite, for which we had sufficient evidence to incorporate into the model.^10^ Omitting these complications and disabilities could underestimate the burden of snakebites and the impact of improving access to antivenom in Lao PDR. This constraint underscores the need for further research to capture the broader spectrum of snakebite-related disabilities in Lao PDR. | Discussion, Limitations, Lines 411-425  Secondly, while snakebite envenoming can result in multiple physical and mental complications and disabilities, such as secondary infections, surgical complications, acute kidney injuries, post-traumatic stress disorder, or anxiety,^35,36^ our model included only amputations, as they are the only disability of snakebite, for which we had sufficient evidence to incorporate into the model.^10^ Severe anaphylactic shock due to antivenom was also not modeled separately. This simplification may underestimate some harms and costs associated with antivenom treatment. However, severe fatal reactions to antivenom are expected to be uncommon based on currently available evidence.^30^ Thus, we adopted a conservative modeling approach and acknowledge that excluding additional complications and long-term sequelae may underestimate the overall burden of snakebite and the potential benefits of improving access to antivenom in Lao PDR. Incorporating these complications would likely increase the estimated burden of snakebite and OOP expenditures, particularly in rural populations, and may further widen the observed urban–rural inequities. This constraint underscores the need for further research to capture the broader spectrum of snakebite-related complications, sequelae, and disabilities in Lao PDR. |

**Comment 7**

Comment 8: With such mortality rates, it's unlikely that 100 % of the victims require AV. Giving treatment to populations with not much to spend is unethical as it leads to high costs and a risk of side effects (anaphylactic shock) with benefit of the treatment. Response was that it may be only 20-30% of victims with snakebite envenoming actually medically require AV referring to a table in the appendix. I suggest the authors to present this more realistic/medically needed access to AV and see how that works out economically.

Discussion now reads ' training healthcare workers to recognize indications for antivenom'. If authors consider this relevant why then do they keep the baseline assumption that 100% of victims need AV?

**Response 7**

We would like to clarify that our model did not assume that 100% of snakebite victims require antivenom treatment. Antivenom was modeled only for patients with systemic envenoming who met clinical indications for antivenom treatment. This point has already been clarified in the revised Methods section, where we stated that in the hypothetical “full access” scenario, “all snakebite victims with systemic envenoming meeting clinical indications for antivenom treatment receive antivenom.”

The percentages presented in Table S2 represent the estimated proportion of snakebite victims with systemic envenoming requiring antivenom treatment in the model (20-30% of snakebite victims). Under the current access scenario, many patients who clinically require antivenom are unable to receive it due to the limited availability of antivenom. Consequently, the purpose of the “full access” scenario was to estimate the potential impact if all clinically indicated patients could appropriately receive antivenom treatment, rather than assuming unnecessary or indiscriminate antivenom use. Therefore, the “full access” scenario does not represent universal antivenom administration to all snakebite victims, but rather a scenario in which all patients who medically require antivenom are able to access it appropriately through conventional healthcare facilities. Therefore, we have elaborated in our manuscript to clarify these points. Please see the changes below.

| **Original version** | **Revised version** |
| --- | --- |
| Abstract, Lines 31-35  **Methods:** An extended cost-effectiveness analysis (ECEA) was conducted to evaluate the distributional impact of improving access to antivenom in urban and rural Lao PDR, where all victims with systemic envenoming clinically indicated for antivenom receive antivenom at conventional hospitals, on disease burdens (deaths and disability-adjusted life years [DALYs]) and economic burden (%household OOP expenditure per monthly income). | Abstract, Lines 31-35  **Methods:** An extended cost-effectiveness analysis (ECEA) was conducted to evaluate the distributional impact of improving access to antivenom in urban and rural Lao PDR, where all victims with systemic envenoming clinically indicated for antivenom receive antivenom at conventional hospitals, on disease burdens (deaths and disability-adjusted life years [DALYs]) and economic burden (%household OOP expenditure per monthly income). |
| Author Summary, Lines 54-61  Previous cost-effectiveness analysis highlighted the substantial clinical and economic benefits of hypothetically treating all snakebite envenoming cases in Lao PDR with antivenoms. However, the study did not examine the distributional impacts of antivenom access across urban and rural populations, nor evaluate the potential benefits of financial risk protection. An extended cost-effectiveness analysis (ECEA) was conducted to evaluate the distributional impact of improving access to antivenom in urban and rural Lao PDR, where all victims receive antivenom at conventional hospitals, on disease burdens and economic burden. | Author Summary, Lines 55-63  A previous cost-effectiveness analysis highlighted the substantial clinical and economic benefits of hypothetically treating all snakebite envenoming with clinical indications of receiving antivenoms in Lao PDR with antivenoms. However, the study did not examine the distributional impacts of antivenom access across urban and rural populations, nor did it evaluate the potential benefits of financial risk protection. An extended cost-effectiveness analysis (ECEA) was conducted to evaluate the distributional impact of improving access to antivenom in urban and rural Lao PDR, where all snakebite envenoming patients with clinical indications of receiving antivenoms could receive antivenom at conventional hospitals, on disease burdens and economic burden. |
| Methods, Overall approach, Lines 139-146  Urban and rural areas were defined according to administrative classifications used in national statistics, with urban areas corresponding to provincial capitals and urban districts, and rural areas encompassing populations outside these administrative boundaries.^18,24,25^ This evaluation compared the “current access” scenario (limited access), where only a small proportion of victims received antivenom treatment in conventional healthcare facilities, with an alternative hypothetical “full access” scenario, where all victims were assumed to ideally seek conventional treatment, and all snakebite victims with systemic envenoming meeting clinical indications for antivenom treatment receive antivenom. | Methods, Overall approach, Lines 1156-167  Urban and rural areas were defined according to administrative classifications used in national statistics, with urban areas corresponding to provincial capitals and urban districts, and rural areas encompassing populations outside these administrative boundaries.^18,24,25^ Approximately 20-30% of snakebite victims have systemic envenoming meeting clinical indications for antivenom treatment.^10^ This evaluation compared the “current access” scenario (limited access), where only a small proportion of victims with systemic envenoming meeting clinical indications for antivenom treatment received antivenom treatment in conventional healthcare facilities due to limited supplies, with an alternative hypothetical “full access” scenario, where all victims were assumed to ideally seek conventional treatment, and all snakebite victims with systemic envenoming meeting clinical indications for antivenom treatment were assumed to receive antivenom at the conventional healthcare facilities, rather than universal administration to all snakebite victims. |

**Comment 8**

Comment 19: Mortality rate = 3% with the current poor access to AV which demonstrates not many victims actually need AV as there's not more than 3% to be gained. REsponse = that there is a difficulty in reporting mortality and 3% may be an underestimate as patients prefer to stay home to die. This kind of contradicts with the given that mortaiity is the easiest and most reliable outcome measure in hospital record keeping. And if mortality would be seriously high; you would have a higher number in hospital even if some patient manage to get themselves discharged to die at home. So, agree, the mortaility rate may be 4 % or maybe even 5% but it's still unlikely that all snakebite victims need AV.

**Response 8**

We agree that mortality is generally considered one of the most objective and reliable clinical outcomes. However, in the context of snakebite envenoming in Lao PDR, mortality estimates are largely derived from hospital-based data and may not fully capture deaths occurring outside the formal healthcare system. In rural settings, some snakebite victims may never reach healthcare facilities because of geographical barriers, delayed care-seeking, or reliance on traditional healers. In addition, some severely ill patients may be discharged home due to cultural preferences for dying at home, resulting in deaths that are not captured in hospital mortality statistics. Therefore, we believe that the currently reported mortality rates may still underestimate the true burden of snakebite-related mortality in Lao PDR. We have added to our Limitations that “Fourthly, mortality estimates in this study were primarily derived from hospital-based data and may not fully capture deaths occurring outside the formal healthcare system. In rural areas of Lao PDR, some snakebite victims may never reach healthcare facilities because of geographical barriers, delayed care-seeking, or reliance on traditional healers. In addition, some severely ill patients may be discharged home due to cultural preferences for dying at home, resulting in deaths that are not captured in hospital mortality statistics. Therefore, the mortality burden of snakebite envenoming in the Lao PDR may be underestimated in this analysis.^9-12^“

We would also like to clarify that our model did not assume that all snakebite victims require antivenom treatment. As clarified in our response to Comment 7 and in the revised Methods section, antivenom was modeled only for patients with systemic envenoming who met clinical indications for antivenom treatment, representing approximately 20–30% of snakebite victims in the model. Therefore, the “full access” scenario reflects a situation in which all clinically indicated patients are able to appropriately access antivenom treatment, rather than universal administration of antivenom to all snakebite victims. Therefore, we have elaborated on these points in our manuscript. Please see the changes below.

| **Original version** | **Revised version** |
| --- | --- |
| Abstract, Lines 31-35  **Methods:** An extended cost-effectiveness analysis (ECEA) was conducted to evaluate the distributional impact of improving access to antivenom in urban and rural Lao PDR, where all victims with systemic envenoming clinically indicated for antivenom receive antivenom at conventional hospitals, on disease burdens (deaths and disability-adjusted life years [DALYs]) and economic burden (%household OOP expenditure per monthly income). | Abstract, Lines 31-35  **Methods:** An extended cost-effectiveness analysis (ECEA) was conducted to evaluate the distributional impact of improving access to antivenom in urban and rural Lao PDR, where all victims with systemic envenoming clinically indicated for antivenom receive antivenom at conventional hospitals, on disease burdens (deaths and disability-adjusted life years [DALYs]) and economic burden (%household OOP expenditure per monthly income). |
| Author Summary, Lines 54-61  Previous cost-effectiveness analysis highlighted the substantial clinical and economic benefits of hypothetically treating all snakebite envenoming cases in Lao PDR with antivenoms. However, the study did not examine the distributional impacts of antivenom access across urban and rural populations, nor evaluate the potential benefits of financial risk protection. An extended cost-effectiveness analysis (ECEA) was conducted to evaluate the distributional impact of improving access to antivenom in urban and rural Lao PDR, where all victims receive antivenom at conventional hospitals, on disease burdens and economic burden. | Author Summary, Lines 55-63  A previous cost-effectiveness analysis highlighted the substantial clinical and economic benefits of hypothetically treating all snakebite envenoming with clinical indications of receiving antivenoms in Lao PDR with antivenoms. However, the study did not examine the distributional impacts of antivenom access across urban and rural populations, nor did it evaluate the potential benefits of financial risk protection. An extended cost-effectiveness analysis (ECEA) was conducted to evaluate the distributional impact of improving access to antivenom in urban and rural Lao PDR, where all snakebite envenoming patients with clinical indications of receiving antivenoms could receive antivenom at conventional hospitals, on disease burdens and economic burden. |
| Methods, Overall approach, Lines 139-146  Urban and rural areas were defined according to administrative classifications used in national statistics, with urban areas corresponding to provincial capitals and urban districts, and rural areas encompassing populations outside these administrative boundaries.^18,24,25^ This evaluation compared the “current access” scenario (limited access), where only a small proportion of victims received antivenom treatment in conventional healthcare facilities, with an alternative hypothetical “full access” scenario, where all victims were assumed to ideally seek conventional treatment, and all snakebite victims with systemic envenoming meeting clinical indications for antivenom treatment receive antivenom. | Methods, Overall approach, Lines 1156-167  Urban and rural areas were defined according to administrative classifications used in national statistics, with urban areas corresponding to provincial capitals and urban districts, and rural areas encompassing populations outside these administrative boundaries.^18,24,25^ Approximately 20-30% of snakebite victims have systemic envenoming meeting clinical indications for antivenom treatment.^10^ This evaluation compared the “current access” scenario (limited access), where only a small proportion of victims with systemic envenoming meeting clinical indications for antivenom treatment received antivenom treatment in conventional healthcare facilities due to limited supplies, with an alternative hypothetical “full access” scenario, where all victims were assumed to ideally seek conventional treatment, and all snakebite victims with systemic envenoming meeting clinical indications for antivenom treatment were assumed to receive antivenom at the conventional healthcare facilities, rather than universal administration to all snakebite victims. |
