# Supplementary material for: Estimating the distributional impact of improving access to snake antivenom in urban and rural Lao People’s Democratic Republic: An extended cost-effectiveness analysis
Source: PLoS Negl Trop Dis. 2026 Jun 4;20(6):e0014420. doi: 10.1371/journal.pntd.0014420 (PMC13268137; doi:10.1371/journal.pntd.0014420)
Supplement: Peer review history

# Peer review: Decision Letter 0

**DOI:** 10.1371/journal.pntd.0014420.r001

**Authors:** Hatem Kallel

11 Jan 2026

-->PNTD-D-25-01152

Estimating the Distributional Impact of Improving Access to Snake Antivenom in Urban and Rural Lao People's Democratic Republic: An Extended Cost-Effectiveness Analysis

PLOS Neglected Tropical Diseases

Dear Dr. Chaiyakunapruk,

Thank you for submitting your manuscript to PLOS Neglected Tropical Diseases. After careful consideration, we feel that it has merit but does not fully meet PLOS Neglected Tropical Diseases's publication criteria as it currently stands. Therefore, we invite you to submit a revised version of the manuscript that addresses the points raised during the review process.

Please submit your revised manuscript within by Mar 12 2026 11:59PM. If you will need more time than this to complete your revisions, please reply to this message or contact the journal office at plosntds@plos.org. When you're ready to submit your revision, log on to https://www.editorialmanager.com/pntd/ and select the 'Submissions Needing Revision' folder to locate your manuscript file.

Please include the following items when submitting your revised manuscript:

* A letter that responds to each point raised by the editor and reviewer(s). You should upload this letter as a separate file labeled 'Response to Reviewers'. This file does not need to include responses to any formatting updates and technical items listed in the 'Journal Requirements' section below.

* A marked-up copy of your manuscript that highlights changes made to the original version. You should upload this as a separate file labeled 'Revised Manuscript with Track Changes'.

* An unmarked version of your revised paper without tracked changes. You should upload this as a separate file labeled 'Manuscript'.

If you would like to make changes to your financial disclosure, competing interests statement, or data availability statement, please make these updates within the submission form at the time of resubmission. Guidelines for resubmitting your figure files are available below the reviewer comments at the end of this letter.

We look forward to receiving your revised manuscript.

Kind regards,

Hatem Kallel

Academic Editor

PLOS Neglected Tropical Diseases

José María Gutiérrez

Section Editor

PLOS Neglected Tropical Diseases

Shaden Kamhawi

co-Editor-in-Chief

PLOS Neglected Tropical Diseases

orcid.org/0000-0003-4304-636XX

Paul Brindley

co-Editor-in-Chief

PLOS Neglected Tropical Diseases

orcid.org/0000-0003-1765-0002

**Additional Editor Comments:**

In this study, authors intended to estimate the Distributional Impact of Improving Access to Snake Antivenom in Urban and Rural Lao People's Democratic Republic: An Extended Cost-Effectiveness Analysis. This manuscript addresses the public health and economic burden of snakebite envenoming in a resource-limited setting, a highly relevant and critical issue within the scope of PNTD. However, the manuscript, in its current form, requires substantial revisions to meet the expected methodological rigor and clarity.

Major Comments:

Robustness of Input Parameters (Methods, lines 155–158; Limitations, lines 349–352):

The Input Parameters used for the 2023 estimates of snakebite incidence in urban and rural Lao PDR (Table S2) rely heavily on assumptions, expert opinion, and outdated data—some dating back to 2010 and 2013. This represents a major methodological vulnerability, particularly given the rapidly evolving demographic and health‑system context of Lao PDR. The authors should substantially expand their description of the quality, representativeness, and vintage of the “publicly available data” used to populate the model.

Specifically, the manuscript should identify:

• The exact government websites or databases consulted.

• The geographical coverage, sampling frame, and representativeness of the “published literature.”

• The number and profile of “local stakeholders” involved, including their areas of expertise.

• The procedures used to elicit expert opinion (structured interviews, Delphi rounds, group discussions, etc.) and the method of aggregation (consensus, mean, median, or other).

If expert elicitation contributed meaningfully to parameter estimation, the authors should provide concrete examples and a transparent, reproducible description of the elicitation methodology.

Table S3 – Micro‑costing of Hospitalization Costs:

The costing of hospitalization for systemic envenoming appears incomplete. Important complications—such as secondary infections, surgical interventions, and renal replacement therapy—are not included, despite occurring in more than 25% of cases in many settings. Their omission likely leads to a substantial underestimation of true hospitalization costs.

Additionally, the rationale for including six urine analyses is unclear. The authors should clarify the clinical management protocol for snakebite envenoming in Lao PDR, including how diagnostic and monitoring practices vary by severity grade.

Table S7 – Micro‑costing of Other Costs:

It is unclear whether human resource costs (nursing, medical, anesthesiology, pharmacist, etc.) were incorporated. These typically represent a major component of hospital expenditures and should be explicitly addressed.

The authors should also clarify whether inter‑facility transfers or medical evacuations—from rural to urban centers for higher‑level care—were included in the costing framework.

Table S2 – Input Parameters:

Several references cited in Table S2 do not correspond to the reference list or numbering in the main manuscript. This discrepancy must be corrected to ensure traceability and reproducibility.

Table S6 – Antivenom Treatment Costs:

The manuscript should specify whether the costs of managing adverse reactions were applied universally to all envenomed patients receiving antivenom, or only to the expected proportion experiencing reactions.

Definition and Implementation of “Full Access”:

The concept of “full access” requires clearer operationalization. Beyond the provision of antivenom, does it imply the absence of geographic barriers, timely access (e.g., within 6 hours), or fully functional supply chains? Even under idealized conditions, real‑world constraints would likely prevent perfect coverage. The authors should explain how “100% conventional treatment” and “100% antivenom treatment” were implemented in the model, given current treatment rates of only 6.6% (urban) and 3.0% (rural). This discrepancy warrants explicit modeling or, at minimum, a dedicated sensitivity analysis.

Model Limitation – Disability Spectrum:

The statement that “our model included only amputations” substantially understates the limitation. Snakebite envenoming is associated with a broad spectrum of long‑term sequelae—including chronic pain, neurological deficits, psychological trauma, and persistent functional impairment—that may contribute significantly to DALYs. The authors should discuss the likely direction and magnitude of bias introduced by excluding these outcomes. A sensitivity analysis incorporating additional, more prevalent but less severe disabilities would meaningfully strengthen the robustness of the DALY estimates and the interpretation of inequities.

Minor Comments

Use consistent terminology throughout. After first defining “Lao People’s Democratic Republic (PDR),” the manuscript should consistently use “Lao PDR.”

Line 357: “Thirdlly” should be corrected to “Thirdly.”

All abbreviations should be defined at first use in the main text, even if previously defined in the abstract.

Conclusion

This manuscript has the potential to make a meaningful contribution to the field. A comprehensive revision addressing the issues outlined above is strongly recommended.

**Journal Requirements:**

If the reviewer comments include a recommendation to cite specific previously published works, please review and evaluate these publications to determine whether they are relevant and should be cited. There is no requirement to cite these works unless the editor has indicated otherwise.

1) Please upload all main figures as separate Figure files in .tif or .eps format. For more information about how to convert and format your figure files please see our guidelines:

https://journals.plos.org/plosntds/s/figures

2) We have noticed that you have uploaded Supporting Information files, but you have not included a list of legends. Please add a full list of legends for your Supporting Information files after the references list.

3) Please amend your detailed Financial Disclosure statement. This is published with the article. It must therefore be completed in full sentences and contain the exact wording you wish to be published.

1) State the initials, alongside each funding source, of each author to receive each grant. For example: "This work was supported by the National Institutes of Health (####### to AM; ###### to CJ) and the National Science Foundation (###### to AM)."

2) State what role the funders took in the study. If the funders had no role in your study, please state: "The funders had no role in study design, data collection and analysis, decision to publish, or preparation of the manuscript."

3) If any authors received a salary from any of your funders, please state which authors and which funders..

If you did not receive any funding for this study, please simply state: u201cThe authors received no specific funding for this work.u201d

4) Please send a completed 'Competing Interests' statement, including any COIs declared by your co-authors. If you have no competing interests to declare, please state "The authors have declared that no competing interests exist". Otherwise please declare all competing interests beginning with the statement "I have read the journal's policy and the authors of this manuscript have the following competing interests"

**Reviewers' Comments:**

Reviewer's Responses to Questions

**Key Review Criteria Required for Acceptance?**

As you describe the new analyses required for acceptance, please consider the following:

**Methods**

-Are the objectives of the study clearly articulated with a clear testable hypothesis stated?

-Is the study design appropriate to address the stated objectives?

-Is the population clearly described and appropriate for the hypothesis being tested?

-Is the sample size sufficient to ensure adequate power to address the hypothesis being tested?

-Were correct statistical analysis used to support conclusions?

-Are there concerns about ethical or regulatory requirements being met?

Reviewer #1: Method

- Line 157-158 – “In cases where input parameters were unavailable, we engaged local stakeholders to obtain them.” Would estimate be a better word than obtain here?

Reviewer #2: No major new analyses or experiments are required for acceptability.

Reviewer #3: (No Response)

Reviewer #4: The manuscript discusses cost-effectiveness and AV distribution which potentially has important information for policy makers.

I nevertheless have difficulties following the manuscript as it is unclear in the results which data is used from the team’s earlier study on cost-effectiveness treating all snakebite envenoming cases and which data has calculations or data collected for this manuscript only.

With the mortality and morbidity used as number per 100,000 inhabitants, why do the authors think the difference between rural and urban areas is based on AV access? I expect this difference to be based on an increased human/snake interaction in rural areas which is related to occupation/accommodation etc and in numbers per 100,000 inhabitants will therefore never change unless you invest in prevention. More useful approach would be to take the morbidity/mortality per snakebite victim when thinking about inequality. The mortality reduction in rural areas is an overestimate (from 2.79 to 1.27 per 100,000 population) of the impact of AV.

Other questions I have are:

Introduction:

Line 72/73: how essential AV if mortality is already that low and reduction of mortality is only 36% of an already low mortality rate? Does the national guideline recommend treatment with AV for all snakebite victims? Low mortality seems to justify treating only people with specific symptoms/indications.

Line 84: how small?

Line 92 so mortality is how much? If only 4.2% of victims get AV and mortality is low, it again suggests only a subset of patients actually needs AV.

106-113: this paragraph seems repetitive.

119: pleas summarize the findings of the study in ref 20 as this study compares full AV use with current AV use and it’s not clear what was added with this study. Can you in the results make clear which findings are used from ref 20 and which data was collected and analysed anew?

Methods:

139: you don’t want to give AV to everyone; what was the definition of need for antivenom? If not exactly known, estimate around half or so depending on earlier findings how many people developed systemic envenoming versus local signs only?

150 point of care = where?

How did you define urban and rural?

Results

225 CI of this estimate? Original obtained data for this manuscript or used from other publications?

229: where does the 744,077 USD come from?

258: rural areas higher mortality is stated but CI does not demonstrate statistical significance so does not seem to justified to state there’s a significant difference

**********

**Results**

-Does the analysis presented match the analysis plan?

-Are the results clearly and completely presented?

-Are the figures (Tables, Images) of sufficient quality for clarity?

Reviewer #1: Results

- Some of the presentation is a bit confusing, I think because of the different perspectives used, e.g. it is stated that there higher cost per patient in rural areas despite less treatment? Hospital cases in urban areas faced OOP expenditures of 102% income, 169% in rural areas. But then – “Under the current access scenario, snakebite victims in rural areas experienced worse epidemiological outcomes but lower economic burdens compared to urban areas.”

- Given the uncertainty it might be a bit strong to conclude that “Rural areas had higher… household economic burden (+1% OOP expenditures per monthly household income, 95% CrI -4% to +6%), than urban areas.” Similar applies to other interpretations throughout this section, e.g. “Rural areas exhibited higher mortality rates (+1.27 deaths per 100,000 population; 95% CrI -0.95 to +7.09) and greater DALYs lost (+32.14 DALYs per 100,000 population; 95% CrI -24.23 to +193.16) compared to urban areas.”

- “Improving access to antivenom reduced health inequities in deaths and DALYs lost between urban and rural areas. However, it exacerbated health inequities in household economic burden.” Is the latter of this based on OOP of patients or overall incidences of CHE?

- Why is out of pocket cost presented as a percentage per patient? While it is an important measure (presumably particularly for the patient) that should be reported, I would have thought that maybe a measure of the numbers of catastrophic health expenditure cases under each scenario would be the better measure to compare the relative impact across the two groups (or probably CHE per 100,000 population to match the other outcomes presented)? Why have the authors chosen to present the data in this way?

- Why is the change in mortality rate for the rural population so uncertain? This casts doubt on the interpretation of these results, but if just applying the effect of greater access, I’m not sure why the credible interval would be so big? Why would it potentially be higher than the current situation?

Reviewer #2: No additional analyses are required to support the Results as presented.

Reviewer #3: (No Response)

Reviewer #4: The manuscript discusses cost-effectiveness and AV distribution which potentially has important information for policy makers.

I nevertheless have difficulties following the manuscript as it is unclear in the results which data is used from the team’s earlier study on cost-effectiveness treating all snakebite envenoming cases and which data has calculations or data collected for this manuscript only.

With the mortality and morbidity used as number per 100,000 inhabitants, why do the authors think the difference between rural and urban areas is based on AV access? I expect this difference to be based on an increased human/snake interaction in rural areas which is related to occupation/accommodation etc and in numbers per 100,000 inhabitants will therefore never change unless you invest in prevention. More useful approach would be to take the morbidity/mortality per snakebite victim when thinking about inequality. The mortality reduction in rural areas is an overestimate (from 2.79 to 1.27 per 100,000 population) of the impact of AV.

Other questions I have are:

Introduction:

Line 72/73: how essential AV if mortality is already that low and reduction of mortality is only 36% of an already low mortality rate? Does the national guideline recommend treatment with AV for all snakebite victims? Low mortality seems to justify treating only people with specific symptoms/indications.

Line 84: how small?

Line 92 so mortality is how much? If only 4.2% of victims get AV and mortality is low, it again suggests only a subset of patients actually needs AV.

106-113: this paragraph seems repetitive.

119: pleas summarize the findings of the study in ref 20 as this study compares full AV use with current AV use and it’s not clear what was added with this study. Can you in the results make clear which findings are used from ref 20 and which data was collected and analysed anew?

Methods:

139: you don’t want to give AV to everyone; what was the definition of need for antivenom? If not exactly known, estimate around half or so depending on earlier findings how many people developed systemic envenoming versus local signs only?

150 point of care = where?

How did you define urban and rural?

Results

225 CI of this estimate? Original obtained data for this manuscript or used from other publications?

229: where does the 744,077 USD come from?

258: rural areas higher mortality is stated but CI does not demonstrate statistical significance so does not seem to justified to state there’s a significant difference

**********

**Conclusions**

-Are the conclusions supported by the data presented?

-Are the limitations of analysis clearly described?

-Do the authors discuss how these data can be helpful to advance our understanding of the topic under study?

-Is public health relevance addressed?

Reviewer #1: Some changes might be needed if changes are made above.

Reviewer #2: No additional analyses are required to support the conclusions.

Reviewer #3: (No Response)

Reviewer #4: related to more general questions which influence conclusions

**********

**Editorial and Data Presentation Modifications?**

Use this section for editorial suggestions as well as relatively minor modifications of existing data that would enhance clarity. If the only modifications needed are minor and/or editorial, you may wish to recommend “Minor Revision” or “Accept”.

Reviewer #1: NA

Reviewer #2: The manuscript is generally clear and well organized, but a few minor editorial and data-presentation issues should be addressed to improve clarity.

In Table 2 and Figure 1, some reported difference values do not match the arithmetic differences implied by the reported urban and rural point estimates. For example, under current access, the difference in %OOP/monthly household income (hospital only) between rural (87%) and urban (42%) populations is reported as +44%, whereas the arithmetic difference is +45%. Similarly, under full access, the reported difference in mortality rate per 100,000 population does not correspond to the difference implied by the reported urban and rural values (1.47 − 0.25 = +1.22, whereas the table and Figure 1 report +1.27). This inconsistency is also reflected in the corresponding figure 1.

The authors should verify these difference calculations and either correct the reported values or clarify in the table footnotes and figure captions that differences are calculated from unrounded model outputs rather than the rounded point estimates shown.

Apart from these points, no new analyses or data re-analysis are required.

Reviewer #3: (No Response)

Reviewer #4: (No Response)

**********

**Summary and General Comments**

Use this section to provide overall comments, discuss strengths/weaknesses of the study, novelty, significance, general execution and scholarship. You may also include additional comments for the author, including concerns about dual publication, research ethics, or publication ethics. If requesting major revision, please articulate the new experiments that are needed.

Reviewer #1: Thanks for the opportunity to review this paper which presents an extended cost effectiveness analysis to examine the impacts of improved access to antivenom in Lao PDR. The work tackles an important issue both in relation to this specific issue as well as the broader debate of how equity should be incorporated into policy deliberations. I have a few comments for the authors to consider, primarily around the presentation and interpretation of their analysis, as decribed in the sections above

Reviewer #2: This manuscript presents a model-based evaluation of the health and equity impacts of improving access to antivenom for snakebite envenoming, using an extended cost-effectiveness analysis framework. The topic is highly relevant to the field of neglected tropical diseases, as snakebite remains a major but under-addressed cause of preventable morbidity, mortality, and financial hardship in low-resource settings.

The strength of the study is its focus on both health outcomes and equity-related outcomes, including out-of-pocket expenditures and distributional effects between urban and rural populations. The use of an extended cost-effectiveness approach is appropriate for the policy questions being addressed and adds value beyond standard cost-effectiveness analyses. Overall, the modeling framework is appropriate, and the analyses are generally well executed.

The manuscript is clearly written and well structured, and the results are presented in a logical and accessible manner. The tables and figures effectively summarize the main findings, and the discussion places the results in a broader public health and policy context.

The only porblem is limited to relatively minor issues in the reporting of difference values in one table and the corresponding figure. These issues appear to be correctable through verification and clarification rather than additional analyses. They do not undermine the overall conclusions of the study.

The work appears to be original, and there are no concerns related to dual publication, research ethics, or publication ethics. The study relies on secondary data sources, and ethical considerations are appropriately addressed.

Overall, this is a policy-relevant study that fits well within the scope of the journal.

Reviewer #3: (No Response)

Reviewer #4: (No Response)

**********

PLOS authors have the option to publish the peer review history of their article (what does this mean?). If published, this will include your full peer review and any attached files.

If you choose “no”, your identity will remain anonymous but your review may still be made public.

**Do you want your identity to be public for this peer review?**  For information about this choice, including consent withdrawal, please see our Privacy Policy.

Reviewer #1: **Yes:** Blake Angell

Reviewer #2: No

Reviewer #3: **Yes:** LUICE AURTIN JOEL JAMES

Reviewer #4: No

**Figure resubmission:**

While revising your submission, we strongly recommend that you use PLOS’s NAAS tool (https://ngplosjournals.pagemajik.ai/artanalysis) to test your figure files. NAAS can convert your figure files to the TIFF file type and meet basic requirements (such as print size, resolution), or provide you with a report on issues that do not meet our requirements and that NAAS cannot fix.

-->

After uploading your figures to PLOS’s NAAS tool - https://ngplosjournals.pagemajik.ai/artanalysis, NAAS will process the files provided and display the results in the "Uploaded Files" section of the page as the processing is complete. If the uploaded figures meet our requirements (or NAAS is able to fix the files to meet our requirements), the figure will be marked as "fixed" above. If NAAS is unable to fix the files, a red "failed" label will appear above. When NAAS has confirmed that the figure files meet our requirements, please download the file via the download option, and include these NAAS processed figure files when submitting your revised manuscript.-->

**Reproducibility:**

To enhance the reproducibility of your results, we recommend that authors of applicable studies deposit laboratory protocols in protocols.io, where a protocol can be assigned its own identifier (DOI) such that it can be cited independently in the future. Additionally, PLOS ONE offers an option to publish peer-reviewed clinical study protocols. Read more information on sharing protocols at https://plos.org/protocols?utm_medium=editorial-email&utm_source=authorletters&utm_campaign=protocols

-->

# Author comment: Author response to Decision Letter 1

**DOI:** 10.1371/journal.pntd.0014420.r002

17 Feb 2026

Attachment Submitted filename: R1_Author response letter_ECEA Snakebite Lao PDR.docx

# Peer review: Decision Letter 1

**DOI:** 10.1371/journal.pntd.0014420.r003

**Authors:** Hatem Kallel

15 Apr 2026

-->PNTD-D-25-01152R1

Estimating the Distributional Impact of Improving Access to Snake Antivenom in Urban and Rural Lao People's Democratic Republic: An Extended Cost-Effectiveness Analysis

PLOS Neglected Tropical Diseases

Dear Dr. Chaiyakunapruk,

Thank you for submitting your manuscript to PLOS Neglected Tropical Diseases. After careful consideration, we feel that it has merit but does not fully meet PLOS Neglected Tropical Diseases's publication criteria as it currently stands. Therefore, we invite you to submit a revised version of the manuscript that addresses the points raised during the review process.

Please submit your revised manuscript within by May 15 2026 11:59PM. If you will need more time than this to complete your revisions, please reply to this message or contact the journal office at plosntds@plos.org. When you're ready to submit your revision, log on to https://www.editorialmanager.com/pntd/ and select the 'Submissions Needing Revision' folder to locate your manuscript file.

Please include the following items when submitting your revised manuscript:

* A letter that responds to each point raised by the editor and reviewer(s). You should upload this letter as a separate file labeled 'Response to Reviewers'. This file does not need to include responses to any formatting updates and technical items listed in the 'Journal Requirements' section below.

* A marked-up copy of your manuscript that highlights changes made to the original version. You should upload this as a separate file labeled 'Revised Manuscript with Track Changes'.

* An unmarked version of your revised paper without tracked changes. You should upload this as a separate file labeled 'Manuscript'.

If you would like to make changes to your financial disclosure, competing interests statement, or data availability statement, please make these updates within the submission form at the time of resubmission. Guidelines for resubmitting your figure files are available below the reviewer comments at the end of this letter.

We look forward to receiving your revised manuscript.

Kind regards,

Hatem Kallel

Academic Editor

PLOS Neglected Tropical Diseases

José María Gutiérrez

Section Editor

PLOS Neglected Tropical Diseases

Shaden Kamhawi

co-Editor-in-Chief

PLOS Neglected Tropical Diseases

orcid.org/0000-0003-4304-636XX

Paul Brindley

co-Editor-in-Chief

PLOS Neglected Tropical Diseases

orcid.org/0000-0003-1765-0002

**Journal Requirements:**

If the reviewer comments include a recommendation to cite specific previously published works, please review and evaluate these publications to determine whether they are relevant and should be cited. There is no requirement to cite these works unless the editor has indicated otherwise.

**Reviewers' Comments:**

Reviewer's Responses to Questions

**Key Review Criteria Required for Acceptance?**

As you describe the new analyses required for acceptance, please consider the following:

**Methods**

-Are the objectives of the study clearly articulated with a clear testable hypothesis stated?

-Is the study design appropriate to address the stated objectives?

-Is the population clearly described and appropriate for the hypothesis being tested?

-Is the sample size sufficient to ensure adequate power to address the hypothesis being tested?

-Were correct statistical analysis used to support conclusions?

-Are there concerns about ethical or regulatory requirements being met?

Reviewer #1: In the response, the authors describe their approach as fitting within an 'ECEA' framework given they do not directly model catastrophic expenditure. This is not refleced in the paper but I think the manuscript could benefit from a more precise description of how the ECEA framework has been applied in this study (particularly around the OOP). While I do not think a traditional ICER is necessarily required for an ECEA, the authors may wish to clarify how this analysis relates to their prior CEA and which components of a full ECEA are, and are not, captured here.

Reviewer #2: (No Response)

Reviewer #4: (No Response)

**********

**Results**

-Does the analysis presented match the analysis plan?

-Are the results clearly and completely presented?

-Are the figures (Tables, Images) of sufficient quality for clarity?

Reviewer #1: Yes

Reviewer #2: (No Response)

Reviewer #4: (No Response)

**********

**Conclusions**

-Are the conclusions supported by the data presented?

-Are the limitations of analysis clearly described?

-Do the authors discuss how these data can be helpful to advance our understanding of the topic under study?

-Is public health relevance addressed?

Reviewer #1: The interpretation of the OOP figure as a share of monthly expenditure is compared to thresholds (e.g. 10% from the Verguet paper) that tend to refer to annual figures I believe. The presentation of these results could be a bit more nuanced I think particularly as (as the authors indicate in their response), they have not modelled catastrophic expenditure but instead included this as an affordability proxy.

Reviewer #2: (No Response)

Reviewer #4: (No Response)

**********

**Editorial and Data Presentation Modifications?**

Use this section for editorial suggestions as well as relatively minor modifications of existing data that would enhance clarity. If the only modifications needed are minor and/or editorial, you may wish to recommend “Minor Revision” or “Accept”.

Reviewer #1: (No Response)

Reviewer #2: With regards to my commnet "In Table 2 and Figure 1, some reported difference values do not match the arithmeticdifferences implied by the reported urban and rural point estimates. For example, under currentaccess, the difference in %OOP/monthly household income (hospital only) between rural(87%) and urban (42%) populations is reported as +44%, whereas the arithmetic difference is+45%. Similarly, under full access, the reported difference in mortality rate per 100,000population does not correspond to the difference implied by the reported urban and rural values(1.47 − 0.25 = +1.22, whereas the table and Figure 1 report +1.27). This inconsistency is alsoreflected in the corresponding figure 1."

The added note is helpful for explaining small discrepancies that can arise when the urban and rural values shown in the table are rounded for readability, while the reported difference is calculated from the underlying unrounded model outputs. For example, if the true values were 42.4 and 86.6, they could be displayed as 42 and 87, while the difference based on the unrounded values would be 44.2, which could reasonably be reported as 44 rather than 45. In that kind of case, the mismatch is understandable and the rounding explanation is sufficient.

However, that explanation does not seem to resolve my concern about the reported +1.27 difference for the full-access mortality rate per 100,000 population. Based on the displayed values, the arithmetic difference is 1.47 - 0.25 = 1.22. If the displayed values are rounded to two decimal places, then the underlying unrounded urban value should be approximately between 0.245 and 0.255, and the rural value should be approximately between 1.465 and 1.475. That would imply an underlying difference of roughly 1.21 to 1.23, not 1.27. So unlike the smaller discrepancies elsewhere in the table, this one appears too large to be explained by rounding alone.

For that reason, I do not think the added note fully addresses this specific issue. If the reported difference of 1.27 was obtained from a different model summary, such as a separately summarized distribution of rural-urban differences rather than direct subtraction of the displayed point estimates, that should be stated explicitly in the table and figure notes. Otherwise, this value still appears inconsistent with the reported urban and rural estimates.

Reviewer #4: (No Response)

**********

**Summary and General Comments**

Use this section to provide overall comments, discuss strengths/weaknesses of the study, novelty, significance, general execution and scholarship. You may also include additional comments for the author, including concerns about dual publication, research ethics, or publication ethics. If requesting major revision, please articulate the new experiments that are needed.

Reviewer #1: The authors have engaged with the reviewer/editorial suggestions which I think has led to a stronger paper.

Reviewer #2: (No Response)

Reviewer #4: Authors answered the comments from the reviewers in a very detailed way. But there answers were to add some sentences here and there but did not change the way data were analysed and/or presented. Some examples:

- comment 1: estimates rely heavily on assumptions, expert opinion and outdated data. Add procedures used to elicit expert opnion (structured interviews, Delphi rounds) and method of aggregation.

-response was to include the references used which include retrospective studies of 21 victims. And to explain the expert opinion was based on three coauthors. the paper would have been much stronger if the methods to use 'expert opinion' was a more systematic one instead of 3 coauthors sitting together. The three authors are reported to be experienced in the clinical management, but 2 of these 3 are pharmacists and 1 is a Germany based researcher. The assumptions for the manuscript are on Lao Democratic republic.

comment 2: reviewer asks for including complications and long term consequences and response = only amputations included. But assumptions on the rate of complications and long term consequences are not less unreliable than the other assumptions in the work. Now looks as authors prefer to keep the main message; that more access to AV has some impact on mortality but will have more economic impact on rural populations. With complications included, that could give a different result. Risk of anaphylactic shock after AV should also be included.

Comment 8: With such mortality rates, it's unlikely that 100 % of the victims require AV. Giving treatment to populations with not much to spend is unethical as it leads to high costs and a risk of side effects (anaphylactic shock) with benefit of the treatment. Response was that it may be only 20-30% of victims with snakebite envenoming actually medically require AV referring to a table in the appendix. I suggest the authors to present this more realistic/medically needed access to AV and see how that works out economically.

Discussion now reads ' training healthcare workers to recognize indications for antivenom'. If authors consider this relevant why then do they keep the baseline assumption that 100% of victims need AV?

Comment 19: Mortality rate = 3% with the current poor access to AV which demonstrates not many victims actually need AV as there's not more than 3% to be gained. REsponse = that there is a difficulty in reporting mortality and 3% may be an underestimate as patients prefer to stay home to die. This kind of contradicts with the given that mortaiity is the easiest and most reliable outcome measure in hospital record keeping. And if mortality would be seriously high; you would have a higher number in hospital even if some patient manage to get themselves discharged to die at home. So, agree, the mortaility rate may be 4 % or maybe even 5% but it's still unlikely that all snakebite victims need AV.

**********

PLOS authors have the option to publish the peer review history of their article (what does this mean?). If published, this will include your full peer review and any attached files.

If you choose “no”, your identity will remain anonymous but your review may still be made public.

**Do you want your identity to be public for this peer review?**  For information about this choice, including consent withdrawal, please see our Privacy Policy.

Reviewer #1: **Yes:** Blake Angell

Reviewer #2: No

Reviewer #4: No

**Figure resubmission:**

While revising your submission, we strongly recommend that you use PLOS’s NAAS tool (https://ngplosjournals.pagemajik.ai/artanalysis) to test your figure files. NAAS can convert your figure files to the TIFF file type and meet basic requirements (such as print size, resolution), or provide you with a report on issues that do not meet our requirements and that NAAS cannot fix.

-->

After uploading your figures to PLOS’s NAAS tool - https://ngplosjournals.pagemajik.ai/artanalysis, NAAS will process the files provided and display the results in the "Uploaded Files" section of the page as the processing is complete. If the uploaded figures meet our requirements (or NAAS is able to fix the files to meet our requirements), the figure will be marked as "fixed" above. If NAAS is unable to fix the files, a red "failed" label will appear above. When NAAS has confirmed that the figure files meet our requirements, please download the file via the download option, and include these NAAS processed figure files when submitting your revised manuscript.-->

**Reproducibility:**

To enhance the reproducibility of your results, we recommend that authors of applicable studies deposit laboratory protocols in protocols.io, where a protocol can be assigned its own identifier (DOI) such that it can be cited independently in the future. Additionally, PLOS ONE offers an option to publish peer-reviewed clinical study protocols. Read more information on sharing protocols at https://plos.org/protocols?utm_medium=editorial-email&utm_source=authorletters&utm_campaign=protocols

-->

# Author comment: Author response to Decision Letter 2

**DOI:** 10.1371/journal.pntd.0014420.r004

6 May 2026

Attachment Submitted filename: R2_Author response letter_ECEA Snakebite Lao PDR NC.docx

# Editor report: Decision Letter 2

**DOI:** 10.1371/journal.pntd.0014420.r005

**Authors:** Hatem Kallel

28 May 2026

Dear Dr Chaiyakunapruk:

We are pleased to inform you that your manuscript 'Estimating the Distributional Impact of Improving Access to Snake Antivenom in Urban and Rural Lao People's Democratic Republic: An Extended Cost-Effectiveness Analysis' has been provisionally accepted for publication in PLOS Neglected Tropical Diseases.

Before your manuscript can be formally accepted you will need to complete some formatting changes, which you will receive in a follow up email. A member of our team will be in touch with a set of requests.

Please note that your manuscript will not be scheduled for publication until you have made the required changes, so a swift response is appreciated.

IMPORTANT: The editorial review process is now complete. PLOS will only permit corrections to spelling, formatting or significant scientific errors from this point onwards. Requests for major changes, or any which affect the scientific understanding of your work, will cause delays to the publication date of your manuscript.

Should you, your institution's press office or the journal office choose to press release your paper, you will automatically be opted out of early publication. We ask that you notify us now if you or your institution is planning to press release the article. All press must be co-ordinated with PLOS.

Thank you again for supporting Open Access publishing; we are looking forward to publishing your work in PLOS Neglected Tropical Diseases.

Best regards,

Hatem Kallel

Academic Editor

PLOS Neglected Tropical Diseases

José María Gutiérrez

Section Editor

PLOS Neglected Tropical Diseases

Shaden Kamhawi

co-Editor-in-Chief

PLOS Neglected Tropical Diseases

orcid.org/0000-0003-4304-636XX

Paul Brindley

co-Editor-in-Chief

PLOS Neglected Tropical Diseases

orcid.org/0000-0003-1765-0002

***********************************************************

p.p1 {margin: 0.0px 0.0px 0.0px 0.0px; line-height: 16.0px; font: 14.0px Arial; color: #323333; -webkit-text-stroke: #323333}span.s1 {font-kerning: none

# Editor report: Acceptance letter

**DOI:** 10.1371/journal.pntd.0014420.r006

**Authors:** Hatem Kallel

Dear Prof. Chaiyakunapruk,

We are delighted to inform you that your manuscript, "Estimating the Distributional Impact of Improving Access to Snake Antivenom in Urban and Rural Lao People's Democratic Republic: An Extended Cost-Effectiveness Analysis," has been formally accepted for publication in PLOS Neglected Tropical Diseases.

We have now passed your article onto the PLOS Production Department who will complete the rest of the publication process. All authors will receive a confirmation email upon publication.

The corresponding author will soon be receiving a typeset proof for review, to ensure errors have not been introduced during production. Please review the PDF proof of your manuscript carefully, as this is the last chance to correct any scientific or type-setting errors. Please note that major changes, or those which affect the scientific understanding of the work, will likely cause delays to the publication date of your manuscript. Note: Proofs for Front Matter articles (Editorial, Viewpoint, Symposium, Review, etc...) are generated on a different schedule and may not be made available as quickly.

Soon after your final files are uploaded, the early version of your manuscript will be published online unless you opted out of this process. The date of the early version will be your article's publication date. The final article will be published to the same URL, and all versions of the paper will be accessible to readers.

For Research Articles, you will receive an invoice from PLOS for your publication fee after your manuscript has reached the completed accept phase. If you receive an email requesting payment before acceptance or for any other service, this may be a phishing scheme. Learn how to identify phishing emails and protect your accounts at https://explore.plos.org/phishing.

Thank you again for supporting open-access publishing; we are looking forward to publishing your work in PLOS Neglected Tropical Diseases.

Best regards,

Shaden Kamhawi

co-Editor-in-Chief

PLOS Neglected Tropical Diseases

Paul Brindley

co-Editor-in-Chief

PLOS Neglected Tropical Diseases
